# Advancing Quantitative ^31^P NMR Spectroscopy for Reliable Thiol Group Analysis

**DOI:** 10.1021/acsmacrolett.5c00739

**Published:** 2026-01-07

**Authors:** Keven Walter, Dominik P. Hoch, Enrico C. Heyl, Ann-Christin Ranieri, Johanna Hansen, André Dallmann, Hans G. Börner

**Affiliations:** 9373 Humboldt-Universität zu Berlin, Department of Chemistry, Laboratory for Organic Synthesis of Functional Systems, Brook-Taylor-Str. 2, 12489 Berlin, Germany

## Abstract

Accurate thiol quantification is essential for advancing thiol–X-ligation strategies in polymer and materials synthesis. Conventional assays, most notably Ellman’s test, are limited in scope, particularly for hydrophobic or multifunctional thiols. Here, we introduce a straightforward and broadly applicable ^31^P NMR spectroscopy method for thiol quantification, using 2-chloro-4,4,5,5-tetramethyl-1,3,2-dioxaphospholane (TMDP) as a phosphitylation reagent. The approach extends established ^31^P NMR protocols for hydroxyl and carboxyl group analysis to thiols, offering high specificity and stability in readout. The method demonstrates applicability across a wide range of substrates, from small organic molecules to polymeric multi thiols with *M*_n_ up to 8000 g·mol^–1^. Comparative validation against Ellman’s assay and ^1^H NMR spectroscopy reveals superior selectivity and resolution of the TMDP-based ^31^P NMR protocol, particularly for technical-grade thiols, where conventional methods fail to distinguish degradation products. This study establishes the TMDP-enabled ^31^P NMR as a reliable, information-rich tool for thiol quantification, giving simultaneously insights on hydroxy and carboxyl functionality patterns.

## Graphical abstract

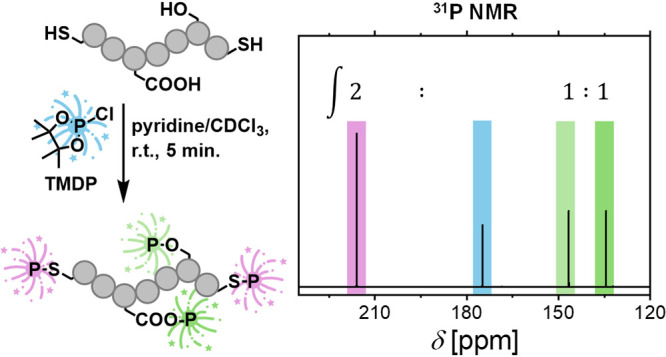

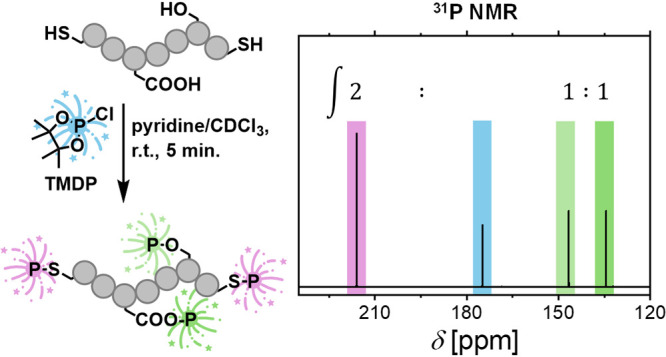

Since the advent of utilizing organic small-molecule strategies in macromolecular synthesis, precise functional group quantification has become essential for advanced polymer and materials synthesis. Among these functionalities, thiols occupy a central role due to their nucleophilicity and ability to form reversible disulfide bonds.^1,2^ The participation takes place in diverse “thiol-X” reactions,^3^ including thiol–ene,^4,5^ thiol–yne,^6^ and thiol–*Michael* ^7^ additions such as thiol–quinones and thiol–acrylate chemistries. These reactions proceed rather selectively, run often under mild conditions, exhibit broad functional group tolerance, and enable the construction of tailored material systems with tunable properties.^8^ Consequently, thiol-functionalized building blocks have emerged as versatile precursors for advanced functional polymer synthesis enabling applications in adhesives,^9–14^ hydrogels,^15,16^ coatings,^17,18^ dendrimers, and amphiphilic systems for drug delivery.^19^ Beyond thiol–X coupling, thiols have been successfully employed in ring-opening polymerization (ROP)^20^ and ring-opening metathesis polymerization (ROMP),^21^ offering degradability, recyclability, and dynamic responsiveness via disulfide exchange.^22^ Emerging strategies using transiently protected mercaptan-alcohols, like thiolactones, allow the synthesis of functional polyesters, precision polymers, and amphiphilic copolymers with tunable self-assembly.^23^ Functional thiols, such as α-lipoic acid derivatives, have recently enabled rapid, room-temperature polymerizations for applications ranging from surgical adhesives to responsive coatings.^24^ Despite the broad utility of thiols, their intrinsic reactivity, particularly the tendency toward air oxidation under ambient conditions, poses challenges for low molecular weight thiols and compromises building block shelf life and reproducibility in material syntheses.^25^ Especially for polymeric systems, accurate thiol quantification is essential, as the degree of functionalization influences mechanical properties, cross-linking density, and long-term stability.^26–28^ Classical methods, including Ellman’s reagent, are widely used, but the analysis is limited to hydrophilic systems and performs with less reliability for polymeric hydrophobic thiols.^29^ As a powerful alternative, ^31^P nuclear magnetic resonance (NMR) spectroscopy allows for the selective derivatization and quantification of hydroxyl and carboxyl groups using phosphitylation reagents such as 2-chloro-1,3,2-dioxaphospholanes (CDP) or 2-chloro-4,4,5,5-tetramethyl-1,3,2-dioxaphospholanes (TMDP).^30^ Benefitting from the 100% natural abundance of ^31^P and a wide chemical shift range, this method has been successfully applied, e.g., for lignin analysis and other complex biomass systems.^31–35^ Given the specificity and robustness, ^31^P NMR might represent a valuable tool for the comprehensive characterization and quality control of thiol-containing polymers.

Here, we present a method for the accurate determination of thiol content in various small molecules and polymers based on derivatization via phosphitylation, followed by readout with ^31^P NMR spectroscopy.

To evaluate the suitability of the described ^31^P NMR method for thiol quantification, 2-mercaptoethanol was selected as a bifunctional model compound, enabling the simultaneous quantification of thiol (−SH) and hydroxyl (−OH) groups (Figure 1A). The procedure followed the standardized protocol for OH pattern analysis as outlined by Meng and co-workers.^32^ The analyte was dissolved in pyridine/CDCl_3_ (1.6:1, v/v), derivatized with either CDP or TMDP (6–10-fold excess), and measured after brief mixing for 30 s. Under these conditions, phosphitylation proceeds rapidly to quantitative conversion, consistent with established ^31^P NMR protocols that have been optimized for sterically demanding biopolymers such as lignin.^32^ Accordingly, neither extension of reaction times nor TMDP excesses beyond 10 equiv affected the thiol quantification results (data not shown). Triphenylphosphine oxide (TPPO) served as internal standard and chromium­(III) acetylacetonate (Cr­(acac)_3_) as relaxation agent. Both phosphitylation reagents generate comparable spectral patterns, with resonances arising from TMDP and the corresponding phosphitylation products appearing slightly downfield shifted (Figure 1B). The derivatized thiols are detected as signals occurring in the range of 200–220 ppm, while the hydroxyl groups are observed in the range of 130–150 ppm. Additionally, the phosphitylation reagents themselves produce signals at 167.27 ppm (CDP) and 174.88 ppm (TMDP), with the hydrolyzed phosphorus species showing signals at 121.10 and 132.20 ppm, respectively. The spectra are aligned to these hydrolyzed phosphorus signals to ensure consistency in comparison.

**Figure 1:**
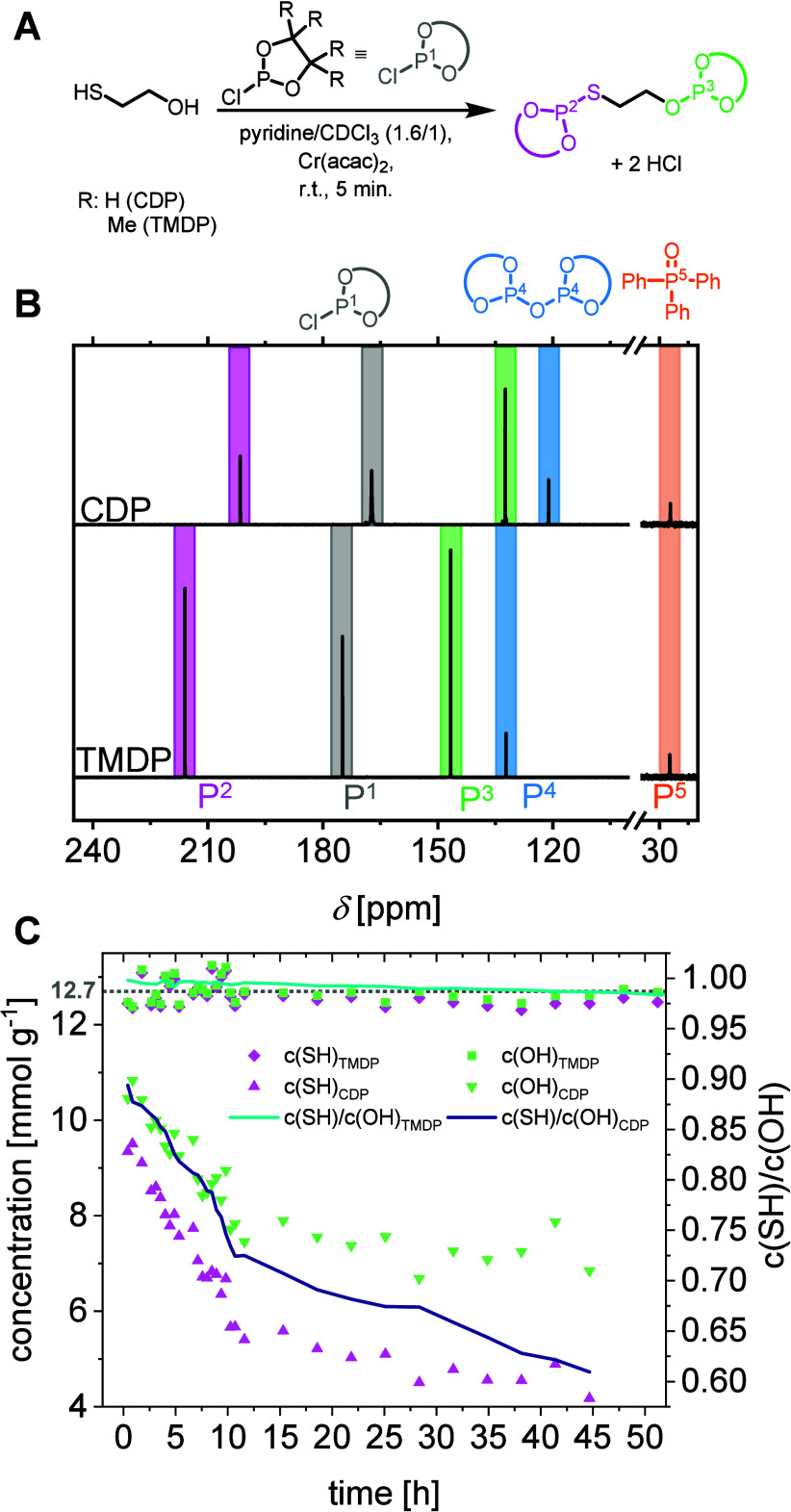
Reaction scheme of the phosphitylation of 2-mercaptoethanol (A) and the corresponding ^31^P NMR spectra of the phosphitylated product recorded 5 min after mixing (B), with the 35–20 ppm region magnified 10-fold to highlight the internal standard. Quantified hydroxyl (−OH) and thiol (−SH) group concentrations obtained from ^31^P NMR analysis (C), with theoretical values indicated by gray dashed line for reference.

Concentrations of −SH and −OH groups were determined from the ^31^P NMR spectra by integrating analyte resonances relative to the triphenylphosphine oxide internal standard (Figure 1C, Equation S3 Supporting Information (SI)). The *c*(OH)/*c*(SH) ratio was determined and plotted as a function of time to monitor signal stability. Samples were analyzed for at least 45 h to assess the potential degradation. Comparative analysis of CDP- and TMDP-based derivatization revealed distinct differences in performance. While CDP exhibited limited accuracy in detecting both–OH and – SH groups, TMDP enabled precise quantification yielding a thiol and hydroxyl content of 12.7 ± 0.5 mmol·g^–1^ (average of the first 3 h, refers to 1 thiol or hydroxyl group per molecule), in excellent agreement with the theoretical value of 12.7 mmol·g^–1^. This result confirms that 99% of the functional groups in low-molecular-weight thiols were successfully detected. By contrast, CDP analysis detected only 82% of hydroxyl and 73% of thiol groups successfully (cf. Table 1). Furthermore, the phosphitylation derivatives formed with CDP undergo rapid degradation during the measurements (Figure 1C). Control experiments with 1-butanol revealed no significant degradation of CDP derivatives over 6 h (Figure S8, SI), indicating that the reduced stability in the 2-mercaptoethanol originates from decomposition of CDP-modified thiol groups, which proceeds considerably faster than the alcohol-derived analogues. This behavior is consistent with the structural differences between both reagents as phosphitylated thiols from CDP lack the steric shielding and hydrophobic microenvironment characteristic to the TMDP derivatives. Probably, reduced hydrophobicity allows access of water or oxygen to the reactive CDP phosphorus centers, thereby accelerating hydrolytic decomposition.^30^ Control experiments demonstrated that even trace amounts of water (0.1 eq relative to TMDP) impair reagent performance. While TMDP showed more robustness under these conditions, it still revealed only about 80% of the expected thiol and hydroxyl species (Figure S12, SI). Collectively, these results show TMDP is superior to CDP for the phosphitylation of thiol-containing compounds such as 2-mercaptoethanol. Over 97% of functional groups were reliably detected, and the resulting phosphitylated derivatives exhibited excellent stability, with only ∼2% signal loss of detected thiol groups after 72 h (Table S3, SI).

**Table 1:**
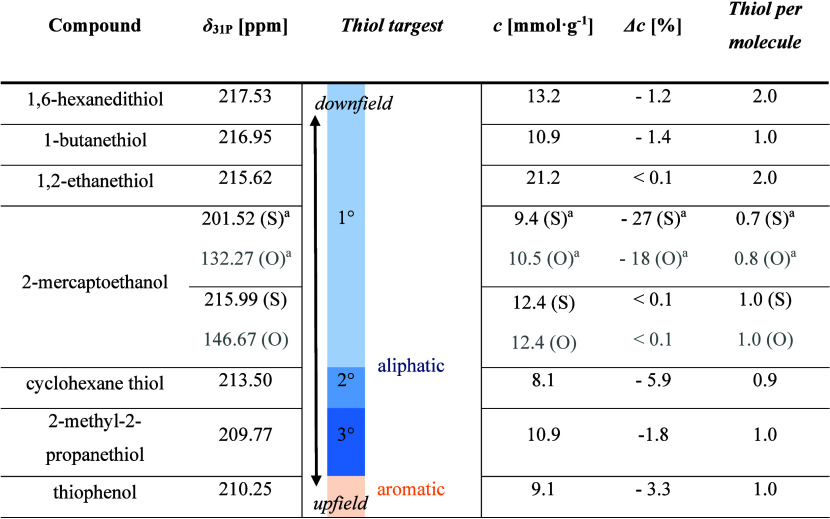
Quantification of Low-Molecular-Weight Thiol Compounds Following TMDP Derivatization and Analysis by ^31^P NMR Spectroscopy, Giving Chemical Shifts (δ_^31^P_) of Resonances, the Calculated Concentration (*c*) and Deviation from Theoretical Concentration (Δ*c*)

a CDP derivatization.

After confirming the reliability of the method using 2-mercaptoethanol as a bifunctional model compound, the applicability to a broader range of substrates was examined. Assay protocol validation and substrate scope were assessed using a series of small molecules featuring primary, secondary, and tertiary thiol groups with both aliphatic and aromatic structures. Following derivatization with TMDP, ^31^P NMR spectroscopy enabled precise quantification of thiol content using TPPO as the internal standard (Table 1 and Section 5.5, SI).

The protocol and procedure proved robustness and broader applicability across aromatic and aliphatic thiol containing substrates (Table 1). It should be noted that the procedure makes use of chemical derivatization and subsequent NMR analysis against TPPO as an internal standard, therefore <3% deviation remains within the expected error of the method. Interestingly, cyclohexanethiol exhibited a higher deviation, underestimating the thiol content by 6%. This might be attributed to the stated material purity as the supplier specified purity by 97%. Surprisingly, the TMDP-based analysis revealed the presence of 0.18 mmol·g^–1^ hydroxyl groups, as evidenced by a distinct ^31^P resonance at 145.15 ppm (Figure S13, SI). The integral corresponded to 2.2% of the total composition. Given that the industrial synthesis of cyclohexanethiol typically proceeds from cyclohexanol, the presence of residual hydroxyl groups suggests incomplete conversion in the thiol functionalization step.^36^ In the case of cyclohexanethiol, this underlines the potentials of the TMDP-based analysis method, giving unambiguous insights into functional compositions of molecules as the different chemical shifts of hydroxyl and thiol groups allow distinguishing and quantification. The sensitivity of the analysis method was investigated using a series of polymers bearing thiol end groups, ideally comprising two to six thiol functionalities per molecule within a molecular weight range of *M*_n_ from 780 to 8000 g·mol^–1^ (Table 2). Accurate quantification is particularly challenging as the polymer compounds show molecular weight distributions, combined with low thiol atom% contents, that make structural assignment difficult.

**Table 2:** Quantification of Macromolecular Thiol Compounds Presented by Theoretical Estimation Based on Ideal Molecular Structure, Classical Thiol Analysis Based on Ellman’s Assay, ^1^H NMR and after Derivatization by ^31^P NMR Giving the Chemical Shift (δ_31P_)­^a^

			average thiol functionalities per molecule		
compound		δ_31P_ [ppm]	theoretical estimation	classical assays^a^ ^,^ ^b^ ^,^ ^c^	^31^P NMR assay
PEG_2000_ dithiol		215.96	2.0	1.9^a^	1.9
PEG_3000_ dithiol		215.96	2.0	1.6^a^	1.50
PEG_8000_ dithiol		215.96	2.0	1.7^a^	1.6 ± 0.1
ETTMP_700_		215.90	3.0	2.1^b^/2.0^c^	2.2 ± 0.1
ETTMP_1300_	aged	215.90	3.0	3.0^b^/2.3^c^	1.5
	fresh			3.0^b^/2.4^c^	1.9
PCL4MP		216.10	4.0	4.7^b^	2.3
DHMP		215.90	6.0	5.5^b^/5.8^c^	4.5
pnBA dithiol		211.02	2		1.65

a Corresponding concentrations are summarized in Table S8 (SI).

b Ellman’s assay.

c Thiol α-CH in ^1^H NMR.

d Ester α-CH in ^1^H NMR.

A set of α-ω-dithiol-functionalized poly­(ethylene glycol)­s (HS-PEG-SH) was analyzed using the TMDP-based ^31^P NMR method. The determined thiol contents were in close agreement with those obtained from Ellman’s assay, the conventional spectroscopic method for thiol quantification (cf. Table 2). Both approaches yielded values in close agreement with the theoretical thiol content for HS-PEG-SH samples with *M*_n_ ≈ 2000 g·mol^–1^ (DP_n_ ≈ 45). For higher molecular weights (up to *M*_n_ ≈ 8000 g·mol^–1^, DP_n_ ≈ 180), small deviations between theoretical and experimental thiol concentrations underline the need for accurate quantification to ensure reliable polymer characterization. For the PEG_8000_ dithiol, reproducibility was evaluated through five independent TMDP derivatization and ^31^P NMR measurements (Section 4.7, SI). The results were highly consistent, giving an average thiol content of 0.20 ± 0.01 mmol·g^–1^, corresponding to 1.6 ± 0.1 thiol groups per molecule, and confirming the high precision of the ^31^P NMR method (cf. Table 2 and Table S8, SI).

While Ellman’s assay proved applicability for water-soluble polymers, its applicability to hydrophobic multifunctional thiols is severely limited due to solubility constraints.^29,37,38^ For more complex substrates, ^1^H NMR spectroscopy was applied to quantify either the α-CH_2_-SH resonances or the α-CH_2_-COOR signals at terminal esters, using octamethylcyclotetrasiloxane (OMCTS) as internal standard (Table 2). As a representative example, the trifunctional ethoxylated trimethylolpropane tri­(3-mercaptopropionate) (ETTMP_700_, *M*_n_ = 700 g·mol^–1^) was investigated. The TMDP-based ^31^P NMR protocol yielded an average of 2.2 ± 0.1 thiol groups per molecule, demonstrating excellent reproducibility across five independent TMDP derivatization and ^31^P NMR measurements (Section 4.7, SI). Although the measured thiol content was lower than the theoretical value, the deviation appears realistic for a technical-grade product. In agreement, ^1^H NMR quantification showed results in a similar range. Integration of the α-CH_2_–SH signals suggested 2.1, while analysis of the α-CH_2_-COOR resonances gave 2.0 thiols per molecule. These minor deviations might potentially arise from overlapping resonances in populated regions of the ^1^H NMR spectra or could indicate partial ester hydrolysis. Indeed, the ^31^P NMR spectra of derivatized ETTMP_700_ further revealed minor contents of hydroxyl (0.27 ± 0.15 mmol·g^–1^) and carboxyl (0.15 ± 0.08 mmol·g^–1^) species, supporting the hypothesis that a fraction of the terminal thiols had undergone hydrolysis (cf. Table S9, SI). These additional functionalities likely explain the minor deviation observed between quantification based on α-CH_2_-SH and α-CH_2_-COOR resonances in the ^1^H NMR spectra. Overall, both ^1^H and ^31^P NMR analysis showed reasonable agreement for ETTMP_700_ although ^31^P NMR quantification offered slightly improved chemical resolution.

Throughout the remaining technical-grade thiols studied, a clear deviation between ^1^H and ^31^P NMR-based quantification was observed (Section 5.6, SI). For PCL4MP, overlapping ester resonances in the oligomer backbone likely caused ^1^H NMR to overestimate the thiol content by approximately 20% compared to the theoretical value, which corresponds to 4.7 thiol groups per molecule (cf. Table 2), whereas the ^31^P NMR protocol yielded only 2.3 thiol groups per molecule. Although the absolute thiol content cannot be independently verified, the ^31^P NMR spectra additionally revealed 0.5 of residual hydroxyl groups per PCL (Figure S24, SI). This observation suggests that the apparent thiol deficiency likely originates from incomplete functionalization of the hydroxyl termini of the PCL star precursor polymer. For DHMP, the theoretical thiol content is 6.0 thiol groups per molecule. ^1^H NMR spectroscopy suggested values of 5.5–5.8 thiol groups per molecule, apparently close to the theoretical expectation. However, ^31^P NMR analysis revealed only 4.5 thiol groups per molecule that were accompanied by 0.4 hydroxyl moieties per DHMP (Figure S25, SI). These findings indicate incomplete esterification of terminal hydroxyl groups by mercaptopropionic acid, resulting in reduced thiol functionality. Collectively, the results show that while ^1^H NMR can yield thiol concentrations near theoretical values, it often overestimates thiol content in multifunctional or technical-grade polymers due to undetected side products. In contrast, ^31^P NMR not only quantifies thiols accurately, but simultaneously reads hydroxyl and carboxyl functionalities, providing a more reliable assessment when discrepancies arise.

This advantage enables ^31^P NMR to identify hydrolysis and/or oxidation products in technical-grade polymers, thereby providing a comprehensive quality profile within a single measurement. Such analytical depth is particularly advantageous for setting stoichiometry in polyaddition/condensation step growth polymerizations or monitoring quality control. For instance, in the trithiol ethoxylated trimethylolpropane tri­(3-mercaptopropionate) (ETTMP_1300_, *M*_n_ = 1300 g·mol^–1^), ^31^P NMR analysis revealed subtle differences between fresh and aged batches, corresponding to 1.9 and 1.5 thiol groups per molecule, respectively (cf. Table 2). These included a slight decrease in thiol content of – 0.3 mmol·g^–1^, accompanied by increases of +0.21 mmol·g^–1^ in hydroxyl and +0.3 mmol·g^–1^ in carboxyl functionalities (Table S8 (SI) and Figures S22 and S23, SI). Such variations could be attributed to the oxidative and hydrolytic processes occurring during storage, which remain undetectable by conventional ^1^H NMR analysis.

As a versatile and widely used route to thiol-endfunctional polymers, reversible addition–fragmentation chain transfer (RAFT) polymerization was selected to evaluate the method at realistic polymer samples. Poly­(*n*-butyl acrylate) (pnBA, *Đ*_GPC_ = 1.08) was synthesized by RAFT in a controlled manner, using a bifunctional dithiobenzoate chain transfer agent (CTA) and subsequent CTA aminolysis yielded α,ω-dithiol pnBA telechelic (Section 5.8, SI). ^1^H NMR confirmed quantitative CTA cleavage and revealed an absolute molecular weight of *M*_n,NMR_ = 6620 g·mol^–1^, when accurately calculating the molecular structure (Figure 2A). Quantitative ^31^P NMR analysis determined an average thiol content of 1.65 functionalities per chain (Figure 2B and Section 5.8, SI), indicating a 17.5% reduction compared to the ideally expected degree of functionalization. Interestingly, this reproducible value is consistent with inherent RAFT process statistics.^39,40^ Considering the CTA:initiator ratio of 2:0.2 and assuming an initiator half-life time of τ_1/2[AIBN]_ in dioxane with ∼3 h at 70 °C,^41^ a decomposition of ∼75% AIBN could be roughly estimated over 6 h polymerization time. This would correlate with a maximum loss of ∼15% chains and agrees with the found thiol content within the general 3% error of the method. Minor disulfide formation might contribute to the reduced thiol-content. However, GPC trace shows only slight tailing at the high molecular weight region and the absence of a distinct peak at twice *M*_n_ suggested predominantly initiator-derived termination pathways (Figure S39, SI). While ^1^H NMR fails to provide reliable thiol quantification due to severe signal overlap and the lack of well-resolved end-group resonances, the ^31^P NMR method enables direct and quantitative assessment of effective thiol functionality (cf. Figure 2A,B), which is particularly valuable for RAFT-derived polymers prone to partial end-group loss.

**Figure 2:**
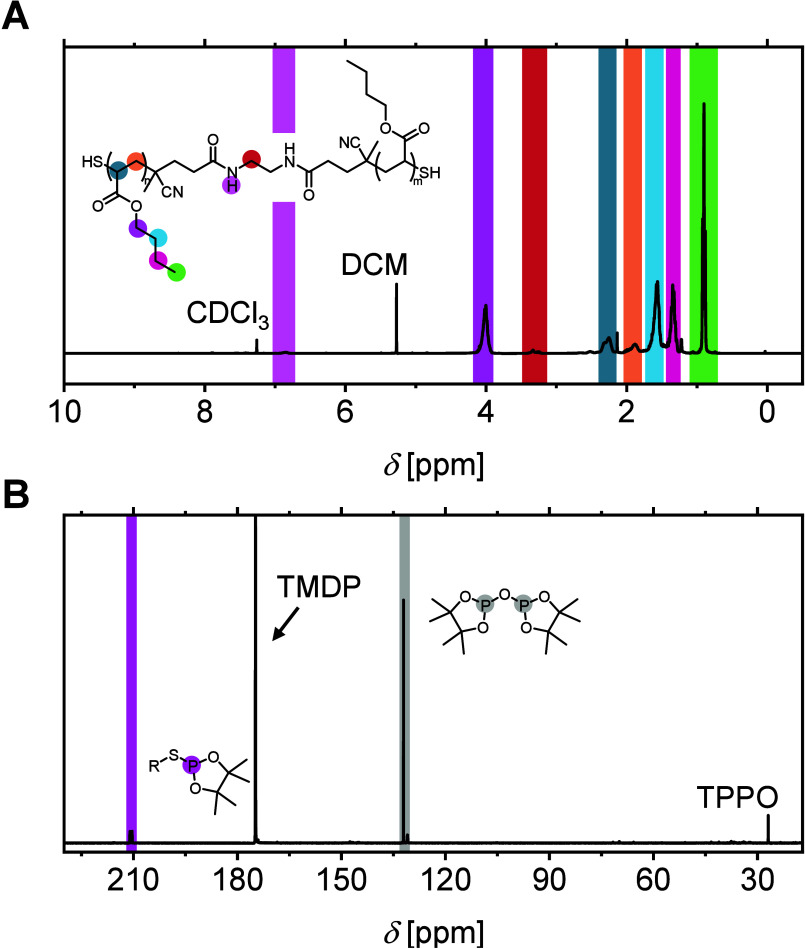
Comparative analysis of RAFT-derived pnBA dithiol by (A) ^1^H NMR in CDCl_3_ and (B) quantitative ^31^P NMR spectrum after TMDP derivatization using triphenylphosphine oxide (TPPO) as internal standard in pyridine/CDCl_3_ (1.6:1, v/v).

In summary, a straightforward method for thiol quantification based on TMDP derivatization and ^31^P NMR spectroscopy was established, extending reliable protocols for hydroxyl analysis. The approach enables accurate quantification of primary, secondary, aliphatic, aromatic, and polymeric multithiols, with typical deviations below 3% and shows for water-soluble PEG dithiols excellent agreements with the Ellman’s assay. Beyond thiol quantification, the method analyzes hydroxyl and carboxyl groups providing insights into oxidative and hydrolytic side products and enabling a comprehensive quality assessment in a single measurement. The method proved robust for small molecules and polymers up to *M*_n_ ≈ 8000 g·mol^–1^, including RAFT-derived polymers, enabling direct assessment of effective thiol end-group functionality. Collectively these findings position ^31^P NMR spectroscopy as a rapid, reliable, and broadly applicable tool for functional group analysis in polymer and materials chemistry.

## Supplementary Material



## Notes

Experimental details, procedures for ^1^H and ^31^P NMR-based quantification, additional NMR spectra, UV/vis data (Ellman’s assay), kinetic stability analyzes, supplementary tables, figures, and supporting references (PDF)
