# Supplementary material for: Advancing Quantitative 31P NMR Spectroscopy for Reliable Thiol Group Analysis
Source: ACS Macro Lett. 2026 Jan 7;15(1):208–14. doi: 10.1021/acsmacrolett.5c00739 (PMC12825386; doi:10.1021/acsmacrolett.5c00739)
Supplement: Supplementary file 1 [file mz5c00739_si_001.pdf]

Supporting Information

Advancing Quantitative ^{31}P NMR Spectroscopy for Reliable Thiol Group Analysis

Keven Walter, Dominik P. Hoch, Enrico C. Heyl, Ann-Christin Ranieri, Johanna Hansen, André Dallmann and Hans G. Börner*

To whom correspondence should be addressed

Prof. Dr. Hans Börner
Humboldt-Universität zu Berlin
Laboratory for Organic Synthesis of Functional Systems
Brook-Taylor-Str. 2, 12489 Berlin
Germany
E-Mail: h.boerner@hu-berlin.de
Web: http://www.boernerlab.de
Phone: +49 (0)30-2093 82852

Table of Contents

1 Chemicals, Solvents, Materials ... 3

2 Instrumentation ... 4

3 Synthesis RAFT Polymerization and Characterization ... 5

3.1 RAFT Agent: 1,2-bis(4-cyano-4-(thiobenzoylthio)pentanoic acid)ethanediamide (BisCTP) ... 5

3.2 RAFT Polymer: poly(n-butyl acrylate) dithiol (pnBA dithiol) ... 6

4 Experimental Procedures ... 7

4.1 Quantification via ^{31}P NMR ... 7

4.2 Ellman's Assay ... 8

4.3 Quantification via ^{1}H NMR ... 9

5 Experimental Data ... 9

5.1 Validation of thiol quantification of 2-mercaptoethanol with CDP over time ... 9

5.2 Validation of thiol quantification of 2-mercaptoethanol with TMDP over time ... 12

5.3 Validation of hydroxyl quantification of 1-butanol with CDP over time ... 15

5.4 Durability testing of quantification under the influence of water ... 18

5.5 Thiol quantification of small molecular compounds ... 18

5.6 Thiol quantification of macromolecular thiol compounds ... 20

5.7 Reproducibility and Precision of Thiol Quantification by ^{31}P NMR ... 28

5.8 Analysis of RAFT dithiol ... 33

6 References ... 36

1 Chemicals, Solvents, Materials

Chemicals

Azobisisobutyronitrile (AIBN, >99%, Acros Organics, Karlsruhe, Germany), *n*-Butylamine (99%, Thermo Fisher Scientific Inc., Dreieich, Germany), *n*-Butylamine (99%, Thermo Fisher Scientific Inc., Dreieich, Germany), Ellman's reagent (5,5′-dithiobis(2-nitrobenzoic acid, 99%, Sigma Aldrich, Darmstadt, Germany), L-cysteine (99%, Sigma Aldrich, Darmstadt, Germany), 2-mercaptoethanol (> 99%, abcr GmbH, Karlsruhe, Germany), 1-butanethiol (98%, abcr GmbH, Karlsruhe, Germany), 2-methyl-2-propanethiol (99%, abcr GmbH, Karlsruhe, Germany), chromium(III) acetylacetonate, ($Cr(acac)_3$, > 98%, Tokyo Chemical Industry Co., Ltd., Eschborn, Germany), cyclohexanethiol (97%, abcr GmbH, Karlsruhe, Germany), dipentaerythritol hexakis(3-mercaptopropionate) (DHMP, technical grade, Bruno Bock Chemische Fabrik GmbH & Co. KG, Marschacht, Germany), dioxan (ROTISOLV® HPLC, Carl Roth GmbH + Co. KG, Karlsruhe, Germany), ethoxylated trimethylolpropane tri(3-mercaptopropionate) ($ETTMP_{700}$, $ETTMP_{1300}$, technical grade, Bruno Bock Chemische Fabrik GmbH & Co. KG, Marschacht, Germany), *N,N′*-dicyclohexylcarbodiimide (Merck AG und Co.Kg, Darmstadt, Germany), octamethylcyclotetrasiloxane (OMCTS, > 98%, Tokyo Chemical Industry Co., Ltd., Eschborn, Germany), poly(ethylene glycol) dithiols (PEG_{2000}, PEG_{3000}, PEG_{8000}, Rapp Polymere GmbH, Tübingen, Germany), polycaprolactone tetra(3-mercaptopropionate) (PCL4MP, technical grade, Bruno Bock Chemische Fabrik GmbH & Co. KG, Marschacht, Germany), thiophenol (98%, Sigma Aldrich, Darmstadt, Germany), sodium phosphate hydrate (>99.5%, Sigma Aldrich, Darmstadt, Germany), disodium phosphate dihydrate (>99.5%, Carl Roth GmbH + Co. KG, Karlsruhe, Germany), 2-Chloro-1,3,2-dioxaphospholane (CDP, 97%, Sigma Aldrich, Darmstadt, Germany), 2-Chloro-4,4,5,5-tetramethyl-1,3,2-dioxaphospholane (TMDP, 95%, Sigma Aldrich, Darmstadt, Germany), 1,2-ethanedithiol (99%, Sigma Aldrich, Darmstadt, Germany), 1,6-hexanedithiol (97%, abcr GmbH, Karlsruhe, Germany), Tetrahydrofuran (THF, HiPerSolv CHROMANORM®, HPLC grade, VWR International GmbH, Darmstadt, Germany).

Dichloromethane, ethyl acetate and methanol (technical grade) were purchased from Stockmeier Chemie GmbH (Berlin, Germany) and distilled prior to use.

Solvents

For NMR spectroscopy $CDCl_3$ (D >99.8%) from Deutero GmbH (Kastellaun, Germany) were used.

Materials

3 Å molecular sieves (Sigma Aldrich, Darmstadt, Germany) were dried at 150 °C under vacuum over the weekend before use.

2 Instrumentation

Nuclear magnetic resonance spectroscopy (NMR) measurements were performed on a Bruker Avance 400 MHz or Avance III 500 MHz spectrometer (Bruker BioSpin GmbH, Rheinstetten, Germany) in the given deuterated solvent.

UV/Vis spectroscopy was performed using a Lambda 950 S UV/Vis/NIR spectrometer (Perkin Elmer, Waltham, USA) and alternatively with a UV-2501PC spectrometer (Shimadzu Deutschland GmbH, Duisburg, Germany) in the range of 200–800 nm.

Fourier-Transform Infrared (FTIR) spectra were recorded on a Vertex 70v spectrometer (Bruker Optik GmbH, Ettlingen, Germany). Blank spectra were collected before and after each measurement.

Ultra-performance liquid chromatography coupled with electrospray ionization mass spectrometry (UHPLC-ESI-MS) was performed using an ACQUITY UPLC® H-Class CM Core system (Waters GmbH, Eschborn, Germany). Detection was carried out using a photodiode array (PDA) detector (190–800 nm) and a QDa mass detector. Chromatographic separation was achieved using a BEH C18 VanGuard™ precolumn (110 Å, 1.7 µm, 5 × 2.1 mm i.d.) in combination with a BEH C18 analytical column (130 Å, 1.7 µm, 5 × 2.1 mm i.d.; both from Waters). Mixtures of water (0.1% formic acid, v/v) and acetonitrile (0.1% formic acid, v/v) were used as mobile phases at a flow rate of 0.5 $mL \cdot min^{-1}$.

Gel permeation chromatography (GPC) measurements were performed on an Eco-SEC system (HLC-8320 GPC, Tosoh, Griesheim, Germany) with UV and RI detection. Tetrahydrofuran (THF, HiPerSolv CHROMANORM®, VWR Chemicals, Dresden, Germany) was used as the eluent. SDV columns (1000 Å, 5 µm; 100 000 Å, 5 µm; 1 000 000 Å, 5 µm; PSS, Mainz, Germany) were applied. Molar masses and dispersities were calculated relative to polystyrene standards (Agilent Technologies: Mp = 580, 4730, 12 980, 19 920, 110 000 $g \cdot mol^{-1}$; Macherey-Nagel: Mp = 1060, 2950, 9200, 30.3k, 66k, 220k, 514k, 1.95M, 3.04M $g \cdot mol^{-1}$) and polyethylene glycol standards (PSS Polymer Standards Service: Mp = 194, 430, 1030, 2130, 3450, 6530, 11.4k, 25.3k, 44k $g \cdot mol^{-1}$).

3 Synthesis RAFT Polymerization and Characterization

3.1 RAFT Agent: 1,2-bis(4-cyano-4-(thiobenzoylthio)pentanoic acid)ethanediamide (BisCTP)

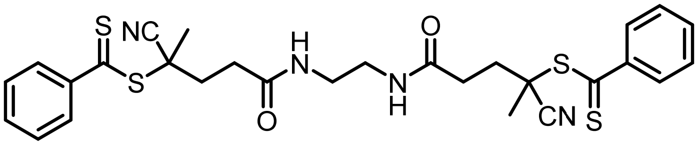

The synthesis of bis(thiobenzoyl) disulfide was carried out according to the procedure reported by Ye *et al.*[1] The synthesis of 4-cyano-4-(thiobenzoylthio)pentanoic acid (CTP) was performed following the literature method described by Thang *et al.*[2] This precursor was subsequently used for the preparation of the bifunctional chain transfer agent (BisCTA) as described below.

4-Cyano-4-(thiobenzoylthio)pentanoic acid (CTP) (5.44 g, 19.49 mmol, 1.00 equiv) was dissolved in dichloromethane (300 mL). *N,N'*-Dicyclohexylcarbodiimide (4.42 g, 21.42 mmol, 1.10 equiv) was then added, and the solution was stirred vigorously at room temperature for 1 h. The reaction mixture was filtered, and the residue was washed with dichloromethane. Subsequently, ethylenediamine (520 µL, 7.79 mmol, 0.40 equiv) was added to the filtrate. The reaction mixture was stirred at room temperature for an additional 2 h and filtered again, with the residue washed with dichloromethane. The filtrate was concentrated *in vacuo* at 40 °C, affording a red oil, which was purified by column chromatography using ethyl acetate as eluent. The target compound BisCTP (2.78 g, 4.77 mmol, 49%) was obtained as a pink crystalline solid.

The analysis data (^{1}H and ^{13}C NMR) for the pnBA dithiol is provided in Section 5.8.

^{1}H NMR (500 MHz, $CDCl_3$): δ [ppm] = 7.82 (t, J = 6.5 Hz, 2H), 7.51 – 7.44 (m, 1H), 7.30 (q, J = 7.5 Hz, 2H), 6.64 (s, 1H), 3.34 (s, 2H), 2.58 – 2.49 (m, 1H), 2.49 – 2.43 (m, 2H), 2.38 – 2.29 (m, 1H), 1.84 (d, J = 5.0 Hz, 2H), 1.20 (d, J = 10.1 Hz, 0H).

^{13}C NMR (126 MHz, $CDCl_3$): δ [ppm] = 222.8, 171.6, 144.6, 133.2, 128.7, 126.8, 118.9, 46.1, 40.1, 34.1, 31.8, 24.3, 14.2.

FT IR (ATR): ν [cm^{-1}] = 1643 (C=O), 2231 (C≡N), 2934 (C-H, aliph.), 3092 (C-H, arom.), 3277 (N-H).

UHPLC-ESI-MS (Gradient (4 min): H_2O + 0.1 % FA : ACN + 0.1 % FA, 6:4 – 1:9, *V*/*V*), λ = 280 nm, pos): t_R [min] = 3.14; calc. *m/z* = 583.81 [M+H]+; found *m/z* = 583.34 [M+H]+.

3.2 RAFT Polymer: poly(n-butyl acrylate) dithiol (pnBA dithiol)

The synthesis of p(nBA) dithiol was carried out following the procedure reported by Schröter *et al.*[3] First, a RAFT polymerization of *n*-butyl acrylate was performed, followed by an ammonolysis to generate free thiol end groups.

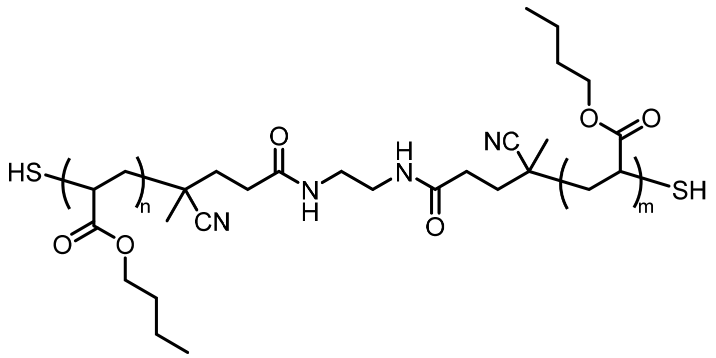

n-Butyl acrylate (nBA) (112.0 mL, 786.46 mmol), 1,2-bis(4-cyano-4-(thiobenzoylthio)pentanoic acid)ethanediamide (BisCTP) (2.29 g, 3.93 mmol), and azobisisobutyronitrile (AIBN) (129.1 mg, 0.79 mmol) were dissolved in dioxane (200 mL) (nBA:CTP:AIBN = 200 : 2: 0.2). The pink solution was degassed under argon for 1 h while stirring and cooling in an ice bath (0 °C). The reaction mixture was then stirred for 6 h at 70 °C in an oil bath under an inert atmosphere, during which the solution turned orange. The reaction was quenched by exposing the mixture to pressurized air. The volatile compounds were removed under reduced pressure. The crude product was precipitated into a mixture of methanol/water (9:1, v/v). After centrifugation, the polymer was reprecipitated into methanol/water (9:1, v/v) two additional times. The product fraction was dissolved in dichloromethane and dried under reduced pressure. BisCTP-*poly*(nBA) (11.9 g) was obtained as an orange, viscous liquid.

GPC (THF): $M_{n,GPC}$ = 4.632 g·mol^{-1}, $M_{w,GPC}$ = 5027 g·mol^{-1}; $Đ$ = 1.08.

BisCTP-*poly*(nBA) (11.9 g, 2.38 mmol, 0.50 equiv.) and tributylphosphine (0.59 mL, 2.38 mmol, 0.50 equiv.) were dissolved in tetrahydrofuran (120 mL). The orange solution was degassed under argon for 1 h while stirring. After this period, *n*-butylamine (0.94 mL, 9.52 mmol, 2.00 equiv.) was added dropwise. The reaction mixture was then stirred at room temperature for 2 h. The resulting greenish-yellow solution was concentrated in vacuo at 30 °C and subsequently poured into five times its volume of cold methanol/water (9:1, v/v), causing the yellowish, viscous polymer to precipitate. After centrifugation, the precipitated polymer was isolated. The precipitation procedure was repeated three times. The resulting colorless polymer solution was dried in vacuo at 30 °C to yield *poly*(nBA) dithiol (7.8 g) as a yellowish, viscous liquid.

The absolute molecular weight of the polymer was determined by ^{1}H NMR spectroscopy (Figure S42) based on integration of the terminal methyl protons of the *n*-butyl acrylate repeat units (δ = 0.90 ppm), using the two amide protons at δ = 6.85 ppm as an internal reference. From this analysis, a degree of polymerization (*DP*) 49 repeat units was obtained.

The analysis data (^{1}H, ^{13}C, ^{31}P NMR and GPC) for the pnBA dithiol is provided in Section 5.8.

^{1}H NMR (500 MHz, $CDCl_3$): δ [ppm] = 6.85 (s, 2H), 4.00 (s, 88H), 3.30 (d, J = 27.8 Hz, 6H), 2.29 (d, J = 28.0 Hz, 43H), 1.87 (s, 28H), 1.73 – 1.48 (m, 148H), 1.35 (s, 109H), 0.90 (t, J = 7.3 Hz, 147H); $M_{n,NMR}$ = 6622 g·mol^{-1}.

^{13}C NMR (101 MHz, $CDCl_3$): δ [ppm] = 174.9, 64.8, 53.8, 41.8, 31.0, 27.7, 24.7, 19.5, 14.1.

FT-IR (ATR): v [cm^{-1}] = 1734 (C=O), 2876 (C-H, aliph.), 2935 (C-H, aliph.), 2961 (C-H, aliph.).

GPC (THF, PS standards): $M_{n,GPC}$ = 5700 g·mol^{-1}; $M_{w,GPC}$ = 6100 g·mol^{-1}; $Đ$ = 1.08.

4 Experimental Procedures

4.1 Quantification via ^{31}P NMR

Sample preparation and NMR analysis were carried out based on the method described by Meng *et al.*[4], with triphenylphosphine oxide (TPPO) used as the internal standard (IS). Additionally, all solutions were stored over a 3 Å molecular sieve to maintain dryness.

The respective model compounds (10-30 mg, corresponding to approximately 0.1-0.3 mmol of phosphitylatable groups) were fully dissolved in 0.45 mL of pyridine/$CDCl_3$ (1.6/1, v/v) at room temperature. Subsequently, the internal standard TPPO (0.1 mL, 0.01 mmol), the relaxation agent Cr(acac)$_3$ (11.4 mg·mL^{-1}, 0.05 mL), and the phosphitylation reagent CDP (0.1 mL, 1.124 mmol) or alternatively TMDP (0.1 mL, 0.629 mmol) were added. The mixture was vortexed for 30 seconds, transferred to an NMR tube, and then measured.

For NMR measurements, the number of scans was chosen to be 128 scans to reach a minimum S/N ratio of 300 for the TPPO signal. The acquisition time of 0.8 s was adjusted to have < 1 Hz fid resolution. A 30° excitation pulse was used in order to facilitate shorter d1 delays and a gated decoupling pulse sequence to ensure proton decoupling but at the same time prevent integration errors due to ^{1}H-^{31}P-NOE effects. The relaxation delay was 10 s and chosen such that any ^{31}P-signal is fully relaxed and the error introduced by relaxation is negligible compared to the typical integration and analysis errors. Deuterated solvents were used throughout to ensure field locking and baseline stability, thereby minimizing integration errors in quantitative ^{31}P NMR measurements.

The resulting spectra were corrected for baseline and phase distortions and referenced to the hydrolysis signal of the phosphitylation reagent. The TPPO signal was then integrated and normalized to a value of 1. Only after this normalization were the other relevant signals integrated. The concentrations of the analytes were subsequently calculated according to Equation S3.

$$c_A = \frac{I_A \cdot n_{IS}}{m_A}$$ (Equation S3)

c_A: concentration of the analyte [$mmol \cdot g^{-1}$]

I_A: NMR integral of the analyte

n_{IS}: amount of substance from IS [mmol]

m_A: sample weight of the analyte [g]

4.2 Ellman's Assay

Preparation of solutions

A 0.1 M, pH = 8 sodium phosphate buffer solution was prepared by dissolving 0.152 g NaH_2PO_4 hydrate and 3.362 g Na_2HPO_4 dihydrate in 200 mL MQ-water. The Ellman's reagent solution was prepared by dissolving Ellman's reagent in buffer with a concentration of 4 $mg \cdot mL^{-1}$.

For the calibration curve a series of diluted cysteine stock solutions was employed. Firstly, cysteine (181.7 mg) was dissolved in 100 mL of buffer. This solution was diluted by a factor of 10 to obtain a 1.5 mM cysteine stock solution. This solution was then further diluted according to Table S1, to obtain solutions with concentrations ranging from 0.10 to 1.25 mM.

Table S 1. Dilution of 1.5 mm cysteine stock solution with buffer (NaH_2PO_4/Na_2HPO4, 0.1 M, pH = 8) to obtain cysteine solution with concentrations ranging from 0.10 to 1.25 mM.

concentration **[mM]**	***volume stock solution*** **[mL]**	***volume buffer*** **[mL]**
1.25	5	1
1.00	4	2
0.75	3	3
0.50	2	4
0.25	1	5
0.10	0.5	7

UV/Vis measurements

The sample preparation was carried out by mixing 1.25 mL buffer, 25 µL of Ellman's reagent solution and 100 µL sample. After shaking this mixture for 15 minutes, the absorption at a wavelength of 412 nm was directly measured. In case of a blank measurement, 100 µL of buffer solution was used instead of a sample. The results for the cysteine calibration curve are shown in Figure S1.

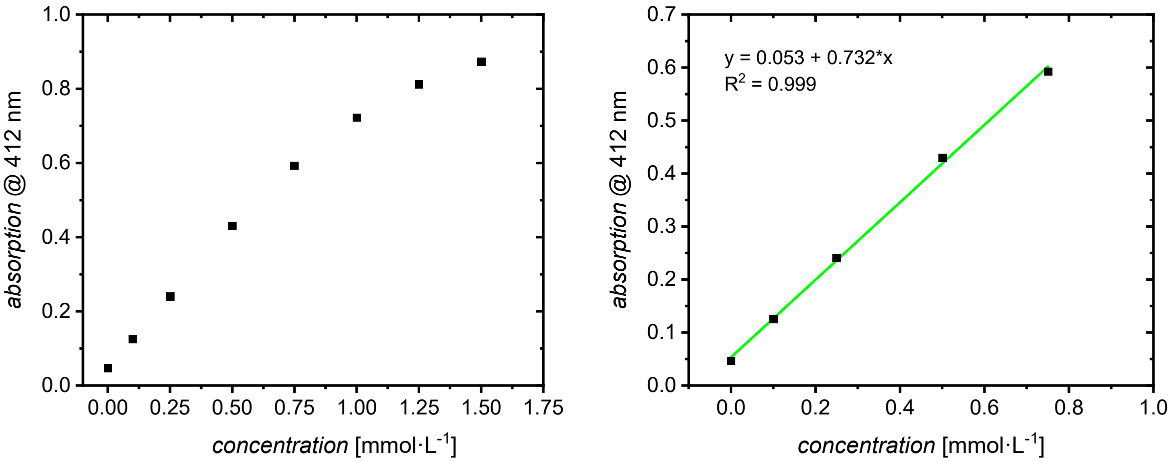

Figure S 1. Calibration curve for UV/Vis absorption measurements of cysteine solutions with different concentrations (left) and linear region with linear fit (right).

The linear region of the calibration curve was determined to be between 0 and 0.75 mM. The thiol concentration of the different samples was calculated according to this linear fit, using the following Equation S4. Here x is the thiol concentration in mmol·L^{-1} and y is the measured absorption at 412 nm.

$$x = \frac{y - 0.053}{0.732} \quad \text{Equation S4}$$

4.3 Quantification via ^{1}H NMR

For the ^{1}H NMR-based quantification, the thiol was dissolved in 600 µL $CDCl_3$. As an internal standard, 50 µL of an OMCTS solution (octamethylcyclotetrasiloxane, 10 µL in 971 µL $CDCl_3$; corresponding to a mixing ratio of 1:97.1) was added.

5 Experimental Data

5.1 Validation of thiol quantification of 2-mercaptoethanol with CDP over time

For the experimental series with CDP, 13.8 mg (0.177 mmol) of 2-mercaptoethanol was used. Representative ^{31}P NMR spectra at the beginning (Figure S2), middle (Figure S3), and end (Figure S4) of the measurement series are shown below.

Table S 2. Kinetic stability analysis of 2-mercaptoethanol after phosphitylation with CDP.

time **[min]**	***integral***		***concentration*** **[mmol·g^{-1}]**	
	**thiol**	**hydroxyl**	**thiol**	**hydroxyl**
25	12.9	14.43	9.35	10.46
50	13.12	14.95	9.51	10.83
75	12.57	14.39	9.11	10.43
100	11.76	13.6	8.52	9.86
125	11.87	13.79	8.60	9.99
150	11.56	13.56	8.38	9.83
175	11.07	13.05	8.02	9.46
200	10.75	12.84	7.79	9.30
225	11.08	13.42	8.03	9.72
250	10.45	12.77	7.57	9.25
275	10.68	13.24	7.74	9.59
300	9.74	12.1	7.06	8.77
325	9.27	11.62	6.72	8.42
350	9.24	11.71	6.70	8.49
375	9.43	11.97	6.83	8.67
400	9.35	12.14	6.78	8.80
425	8.77	11.49	6.36	8.33
450	9.22	12.35	6.68	8.95
475	7.82	10.65	5.67	7.72
500	7.83	10.81	5.67	7.83
525	7.46	10.29	5.41	7.46
550	7.71	10.9	5.59	7.90
575	7.2	10.42	5.22	7.55
600	6.94	10.18	5.03	7.38
625	7.04	10.44	5.10	7.57
650	6.22	9.23	4.51	6.69
675	6.6	10.02	4.78	7.26
700	6.29	9.77	4.56	7.08
725	6.28	10	4.55	7.25
750	6.75	10.86	4.89	7.87
775	5.76	9.45	4.17	6.85

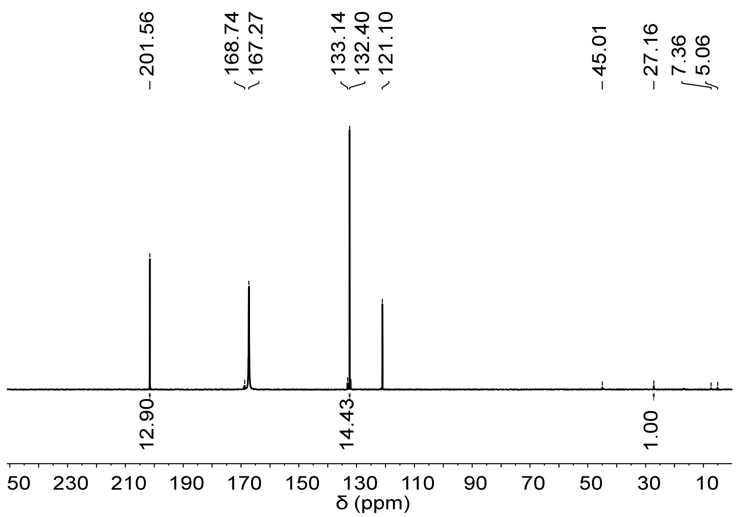

Figure S 2. ^{31}P NMR spectrum of 2-mercaptoethanol phosphitylated with CDP after 25 min in $CDCl_3$/pyridine (1/1.6, v/v) at 162 MHz.

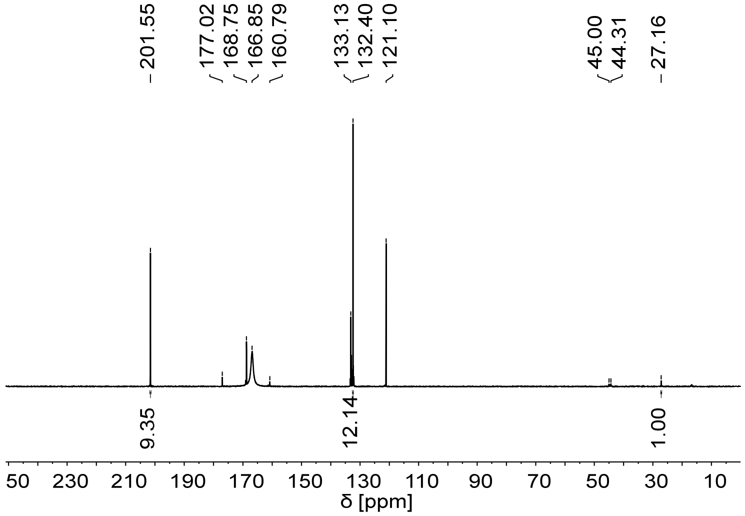

Figure S 3. ^{31}P NMR spectrum of 2-mercaptoethanol phosphitylated with CDP after 400 min in $CDCl_3$/pyridine (1/1.6, v/v) at 162 MHz.

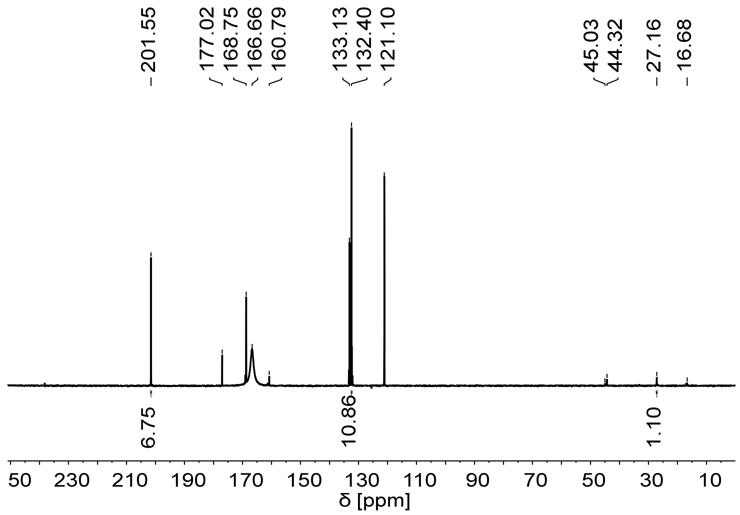

Figure S 4. ^{31}P NMR spectrum of 2-mercaptoethanol phosphitylated with CDP after 775 min in $CDCl_3$/pyridine (1/1.6, v/v) at 162 MHz.

5.2 Validation of thiol quantification of 2-mercaptoethanol with TMDP over time

For the experimental series with TMDP, 16.0 mg (0.205 mmol) of 2-mercaptoethanol was used. Representative ^{31}P NMR spectra at the beginning (Figure S5), middle (Figure S6), and end (Figure S7) of the measurement series are shown below.

Table S 3. Kinetic stability analysis of 2-mercaptoethanol after phosphitylation with TMDP.

time **[min]**	***integral***		***concentration*** **[mmol·g^{-1}]**	
	**thiol**	**hydroxyl**	**thiol**	**hydroxyl**
25	19.91	19.95	12.44	12.47
52	19.76	19.81	12.35	12.38
79	21.35	21.42	13.34	13.39
106	20.94	21.04	13.09	13.15
132	19.52	19.59	12.20	12.24
159	19.85	19.96	12.41	12.48
186	20.09	20.21	12.56	12.63
213	19.81	19.89	12.38	12.43
240	20.78	20.83	12.99	13.02
267	20.49	20.58	12.81	12.86
293	20.74	20.92	12.96	13.08
320	19.8	19.87	12.38	12.42
347	19.3	19.42	12.06	12.14
374	21.82	21.97	13.64	13.73
401	20.21	20.27	12.63	12.67
428	20.43	20.52	12.77	12.83
454	20.31	20.39	12.69	12.74
481	20.16	20.25	12.60	12.66
508	21.08	21.19	13.18	13.24
535	20.46	20.53	12.79	12.83
562	20.79	20.9	12.99	13.06
589	21.01	21.13	13.13	13.21
615	20.14	20.27	12.59	12.67
642	19.83	19.95	12.39	12.47
669	21.84	21.94	13.65	13.71
696	20.22	20.31	12.64	12.69
723	19.48	19.54	12.18	12.21
919	20.16	20.27	12.60	12.67
1114	20.03	20.18	12.52	12.61
1310	20.13	20.28	12.58	12.68
1506	19.79	19.95	12.37	12.47
1701	20.1	20.31	12.56	12.69
1897	19.95	20.15	12.47	12.59
2093	19.83	20.05	12.39	12.53
2289	19.69	19.92	12.31	12.45
2484	19.91	20.17	12.44	12.61

2680	19.91	20.18	12.44	12.61
2876	20.1	20.39	12.56	12.74
3072	19.96	20.29	12.48	12.68
3916	20.06	20.42	12.54	12.76
4335	19.47	19.97	12.17	12.48
10100	18.63	19.29	11.64	12.06

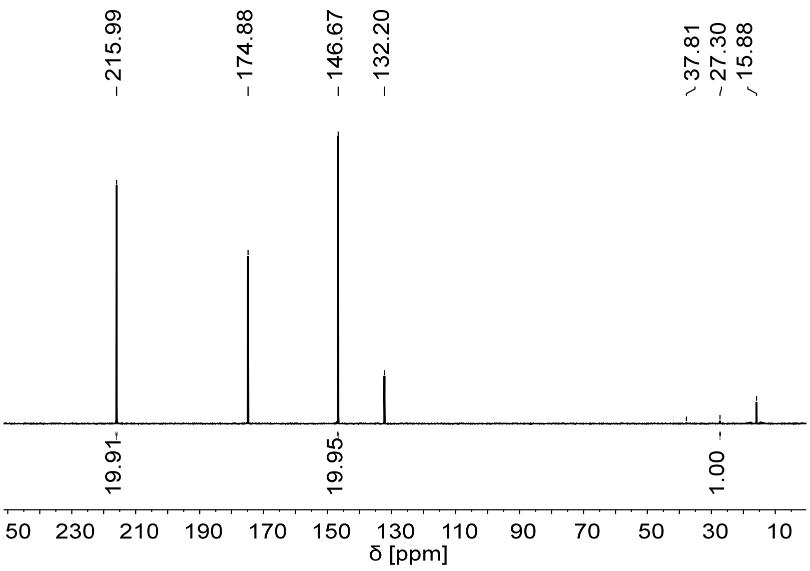

Figure S 5. ^{31}P NMR spectrum of 2-mercaptoethanol phosphitylated with TMDP after 25 min in $CDCl_3$/pyridine (1/1.6, v/v) at 162 MHz.

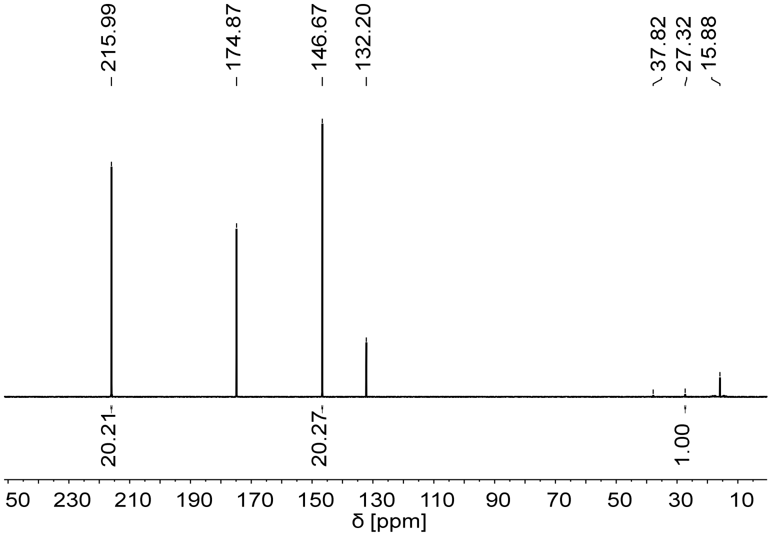

Figure S 6. ^{31}P NMR spectrum of 2-mercaptoethanol phosphitylated with TMDP after 401 min in $CDCl_3$/pyridine (1/1.6, v/v) at 162 MHz.

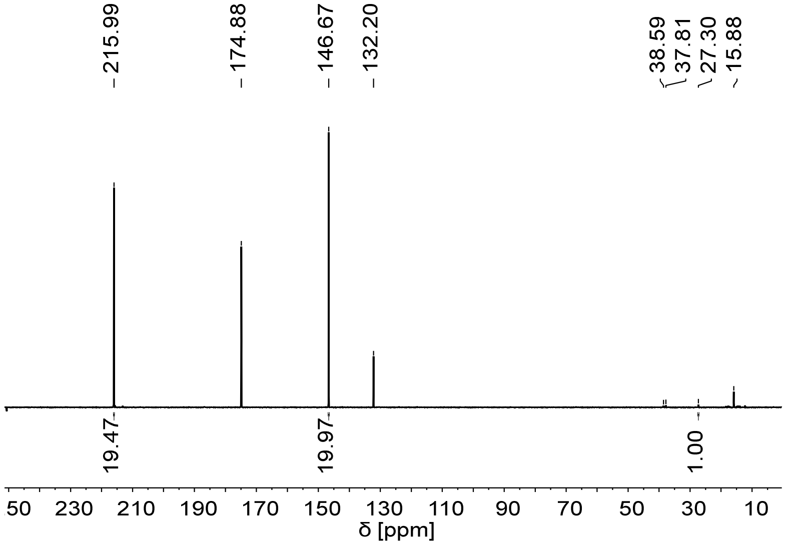

Figure S 7. ^{31}P NMR spectrum of 2-mercaptoethanol phosphitylated with TMDP after 4435 min in $CDCl_3$/pyridine (1/1.6, v/v) at 162 MHz.

5.3 Validation of hydroxyl quantification of 1-butanol with CDP over time

For the experimental series with CDP, 28.9 mg (0.39 mmol) of 1-butanol was used. Representative ^{31}P NMR spectra at the beginning (Figure S9), middle (Figure S10), and end (Figure S11) of the measurement series are shown below.

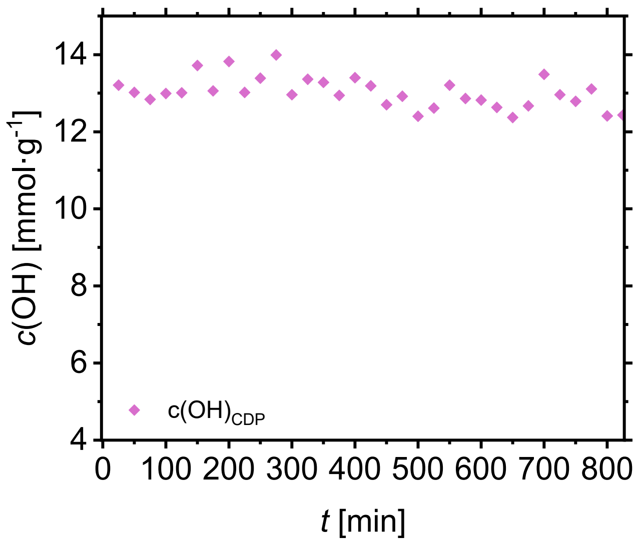

Figure S 8. Quantified hydroxyl (–OH) group concentrations obtained from ^{31}P NMR analysis of 1-butanol with CDP.

Table S 4. Kinetic stability analysis of 1-butanol after phosphitylation with CDP.

time **[min]**	***integral***	***concentration*** **[mmol·g^{-1}]**
	**hydroxyl**	**hydroxyl**
25		13.21
50		13.02
75		12.84
100		12.99
125		13.01
150		13.72
175		13.06
200		13.82
225		13.02
250		13.39
275		13.99
300		12.96
325		13.36
350		13.28
375		12.94
400		13.40
425		13.19
450		12.70
475		12.92
500		12.40
525		12.61
550		13.21
575		12.86
600		12.82
625		12.63
650		12.37
675		12.67
700		13.49
725		12.96
750		12.79
775		13.11
800		12.41
825		12.43

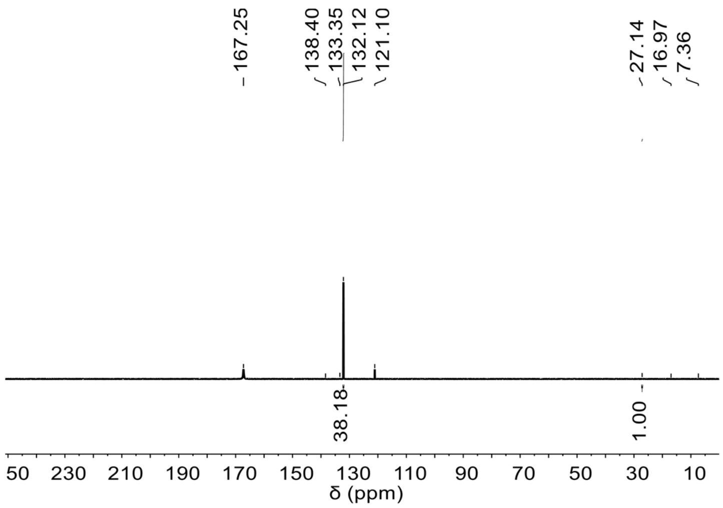

Figure S 9. ^{31}P NMR spectrum of 2-mercaptoethanol phosphitylated with CDP after 25 min in $CDCl_3$/pyridine (1/1.6, v/v) at 162 MHz.

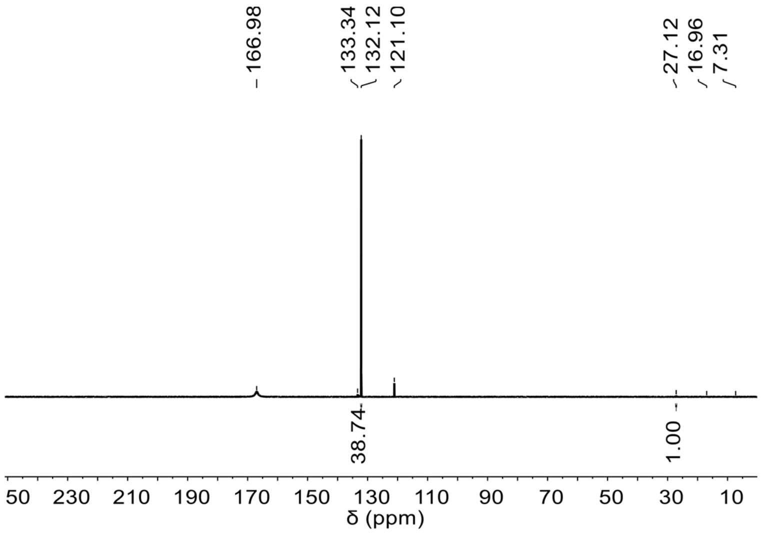

Figure S 10. ^{31}P NMR spectrum of 2-mercaptoethanol phosphitylated with CDP after 400 min in $CDCl_3$/pyridine (1/1.6, v/v) at 162 MHz.

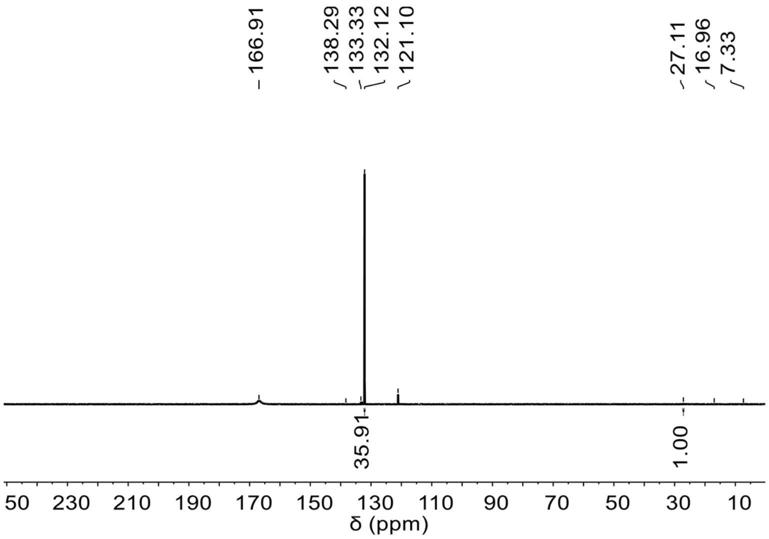

Figure S 11. ^{31}P NMR spectrum of 2-mercaptoethanol phosphitylated with CDP after 825 min in $CDCl_3$/pyridine (1/1.6, v/v) at 162 MHz.

5.4 Durability testing of quantification under the influence of water

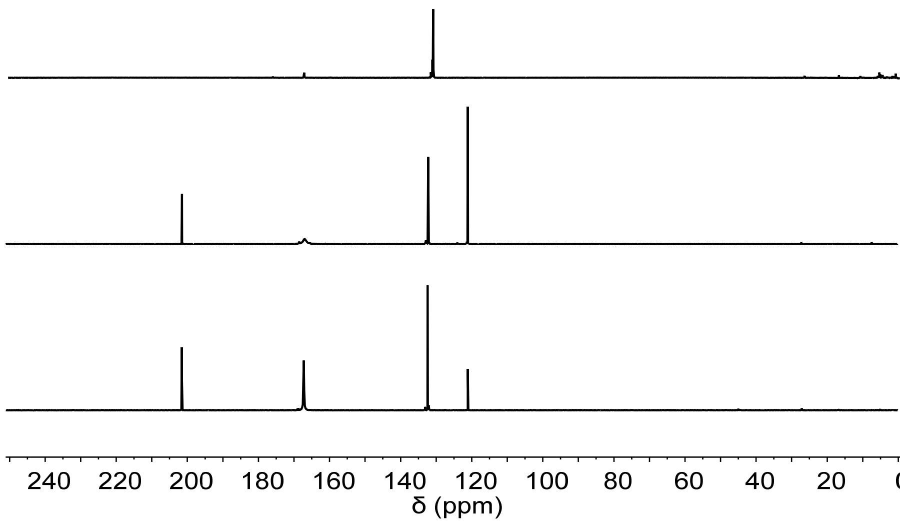

Figure S 12. ^{31}P NMR spectra of 2-mercaptoethanol phosphitylated with TMDP before (bottom) and after addition of water (0.1 eq, middle; 1 eq, top) relative to TMDP in $CDCl_3$/pyridine (1/1.6, v/v) at 162 MHz.

5.5 Thiol quantification of small molecular compounds

Table S 5. Weighed-in amounts and results of the ^{31}P NMR-based quantification method of small molecular compounds.

compound	*m* [mg]	δ_{31P} [ppm]	*Integral*	*c(SH)* [mmol·g^{-1}]	Figure
1-butanethiol	9.2	217.77	10.01	10.9	S9
cyclohexane thiol	14.3	213.52	11.60	8.1	S13
2-methyl-2-propanethiol	9.7	209.77	10.34	10.9	S14
1,2-ethanedithiol	12.1	215.62	26.65	21.2	S15
1,6-hexanedithiol	22.1	217.53	29.88	13.2	S16
thiolphenol	22.6	208.57	20.57	9.1	S17

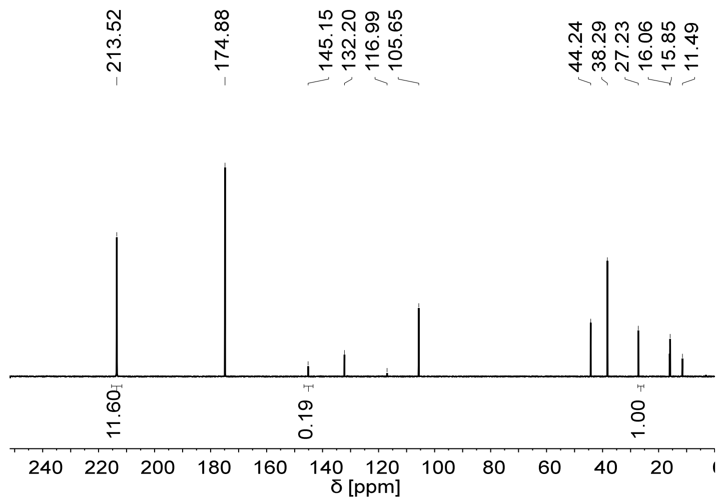

Figure S 13. ^{31}P NMR spectrum of cyclohexane thiol phosphitylated with TMDP in $CDCl_3$/pyridine (1/1.6, v/v) at 162 MHz.

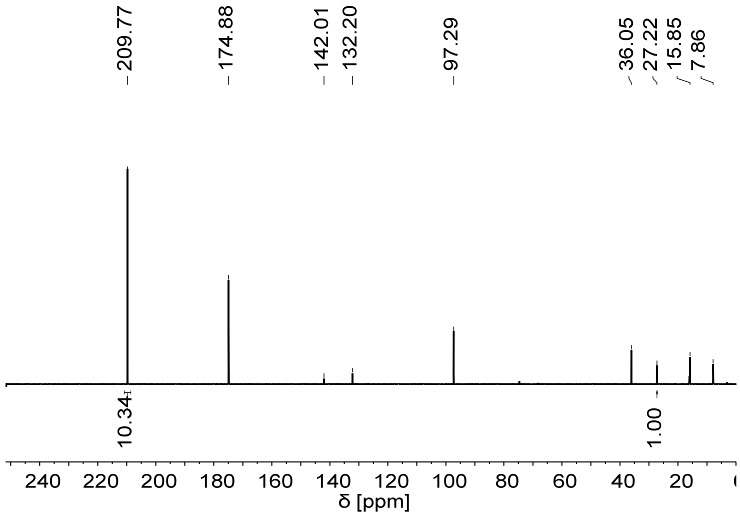

Figure S 14. ^{31}P NMR spectrum of 2-methyl-2-propane thiol thiol phosphitylated with TMDP in $CDCl_3$/pyridine (1/1.6, v/v) at 162 MHz.

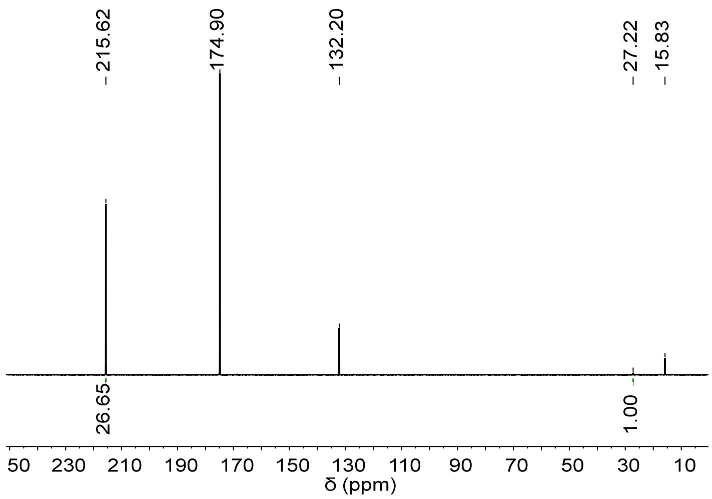

Figure S 15. ^{31}P NMR spectrum of 1,2-ethane thiol phosphitylated with TMDP in $CDCl_3$/pyridine (1/1.6, v/v) at 162 MHz.

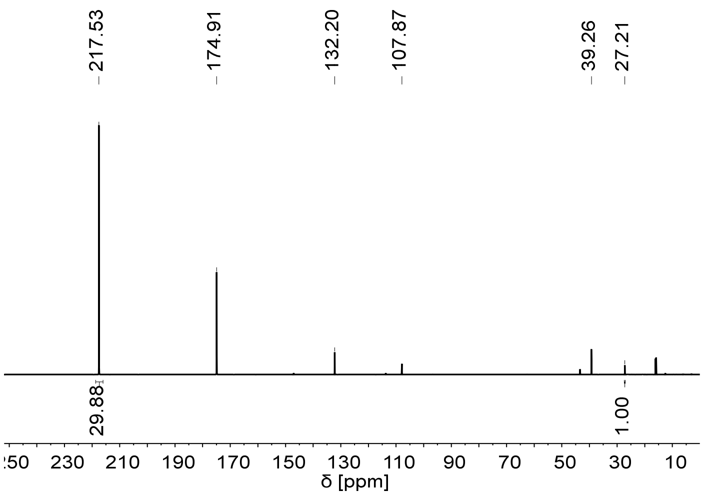

Figure S 16. ^{31}P NMR spectrum of 1-hexane thiol phosphitylated with TMDP in $CDCl_3$/pyridine (1/1.6, v/v) at 162 MHz.

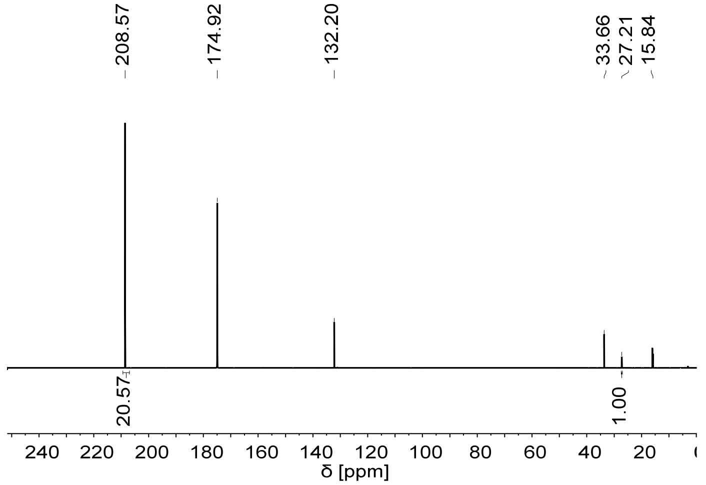

Figure S 17. ^{31}P NMR spectrum of thiolphenol thiol phosphitylated with TMDP in $CDCl_3$/pyridine (1/1.6, v/v) at 162 MHz.

5.6 Thiol quantification of macromolecular thiol compounds

Table S 6. Weighed-in amounts and results of the ^{31}P NMR-based quantification method of macromolecular molecular compounds.

compound		***m*** **[mg]**	δ_{31P} **[ppm]**	***Integral***	***c*(SH) [mmol·g^{-1}]**	**Figure**
PEG_{2000} dithiol		30.2	215.96	1.91	0.94	S18
PEG_{3000} dithiol		12.5	215.96	0.66	0.50	S19
PEG_{8000} dithiol		34.2	215.96	0.80	0.21	S20
$ETTMP_{700}$		33.2	215.90	10.55	3.18	S21
$ETTMP_{1300}$	aged	34.1	215.90	2.71 + 0.59	1.18 + 0.26	S22
	fresh	23.0		5.10	1.47	S23
PCL4MP		32.7	216.10	5.57	1.70	S24
DHMP		33.9	215.90	38.73	5.72	S25

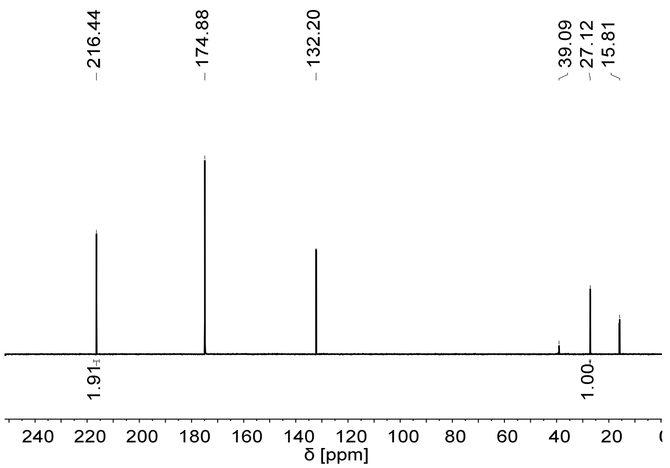

Figure S 18. ^{31}P NMR spectrum of PEG$_{2000}$ dithiol phosphitylated with TMDP in CDCl$_3$/pyridine (1/1.6, v/v) at 202 MHz.

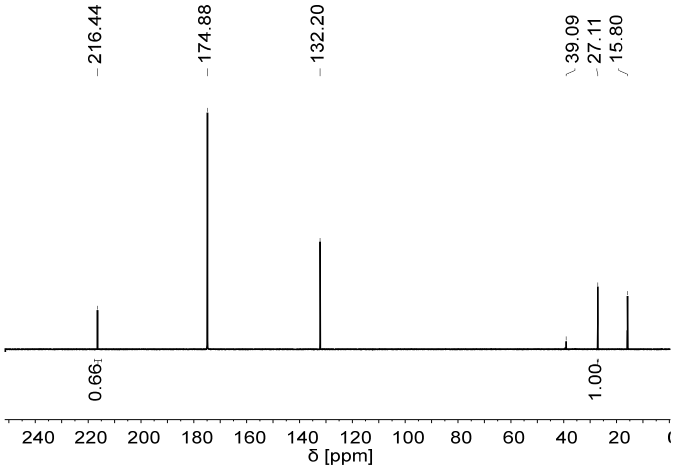

Figure S 19. ^{31}P NMR spectrum of PEG$_{3000}$ dithiol phosphitylated with TMDP in CDCl$_3$/pyridine (1/1.6, v/v) at 202 MHz.

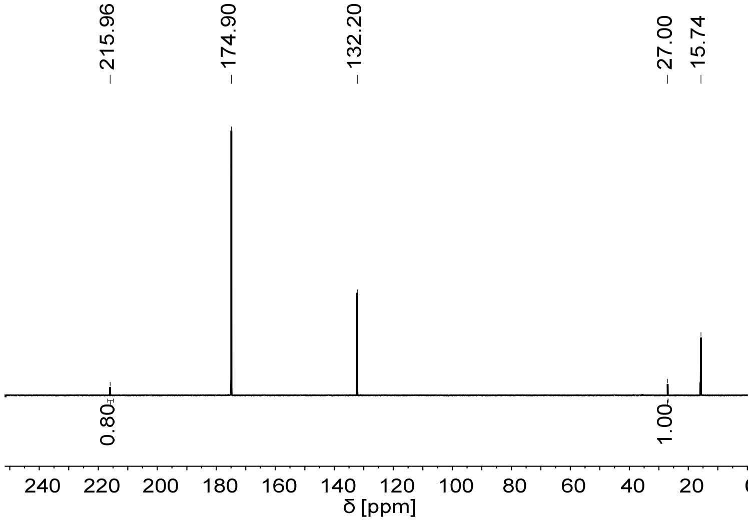

Figure S 20. ^{31}P NMR spectrum of PEG$_{8000}$ dithiol phosphitylated with TMDP in CDCl$_3$/pyridine (1/1.6, v/v) at 202 MHz.

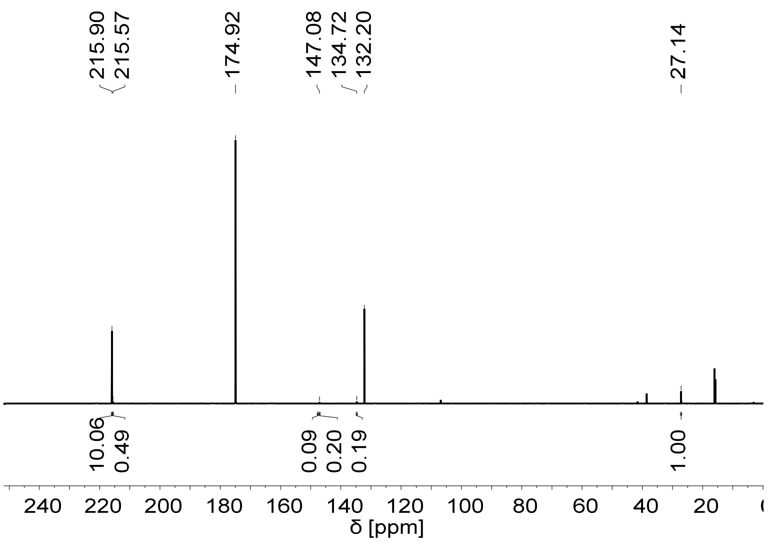

Figure S 21. ^{31}P NMR spectrum of ETTMP700 phosphitylated with TMDP in CDCl3/pyridine (1/1.6, v/v) at 202 MHz.

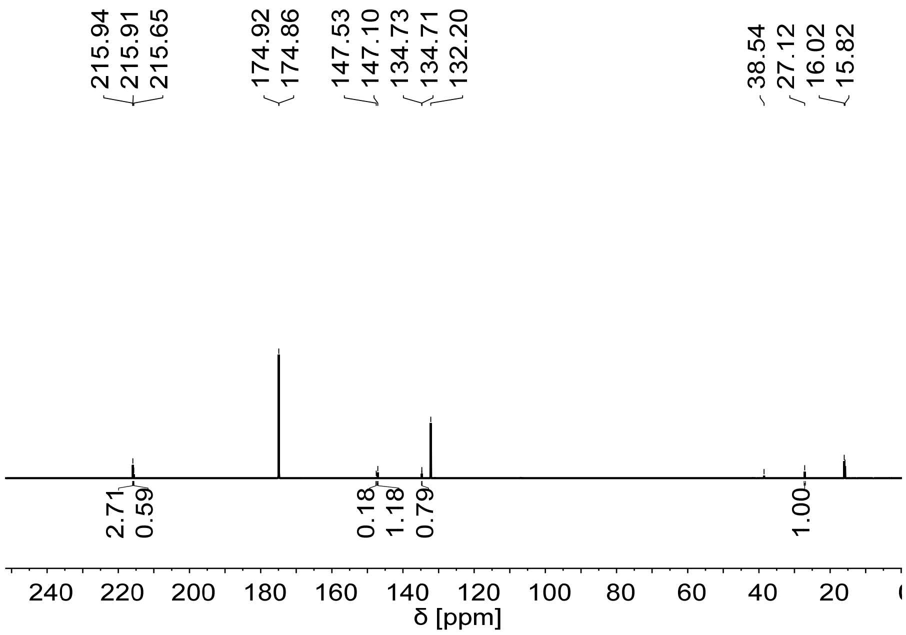

Figure S 22. ^{31}P NMR spectrum of ETTMP1300 (aged) phosphitylated with TMDP in CDCl3/pyridine (1/1.6, v/v) at 202 MHz.

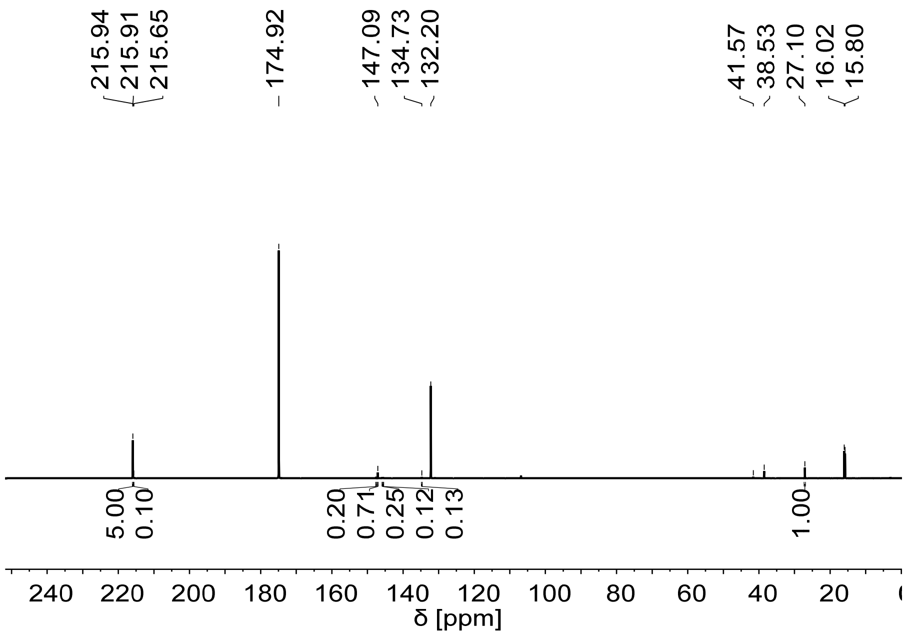

Figure S 23. ³¹P NMR spectrum of ETTMP$_{1300}$ (fresh) phosphitylated with TMDP in CDCl$_3$/pyridine (1/1.6, v/v) at 202 MHz.

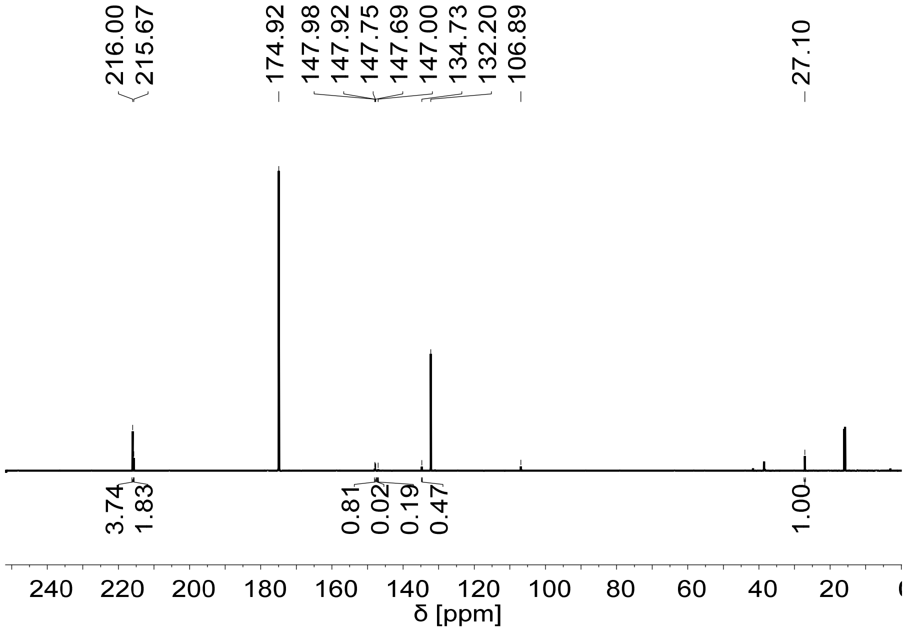

Figure S 24. ³¹P NMR spectrum of PCL4MP phosphitylated with TMDP in CDCl$_3$/pyridine (1/1.6, v/v) at 202 MHz.

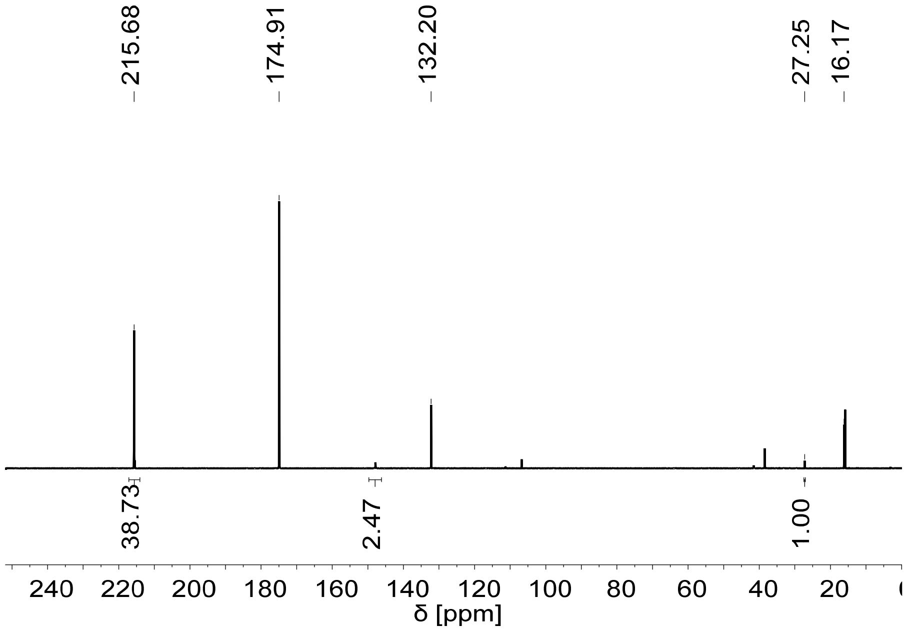

Figure S 25. ^{31}P NMR spectrum of DHMP phosphitylated with TMDP in $CDCl_3$/pyridine (1/1.6, v/v) at 202 MHz.

Table S 7. Weighed-in amounts and results of the ^{1}H NMR-based quantification method of macromolecular molecular compounds.

**compound**		***m*** **[mg]**	***δ***	***Inte-gral***		***Integral***	**Figure**
$ETTMP_{700}$		19.6	4.25	1.37	2.78	1.42	S26
$ETTMP_{1300}$	aged	35.4	4.21	1.37	2.70	2.14	S27
$ETTMP_{1300}$	fresh	30.2	4.21	1.42	2.70	1.67	S28
PCL4MP		37.2	-	-	2.72	3.26	S29
DHMP		41.3	4.12	7.35	2.63	7.29	S30

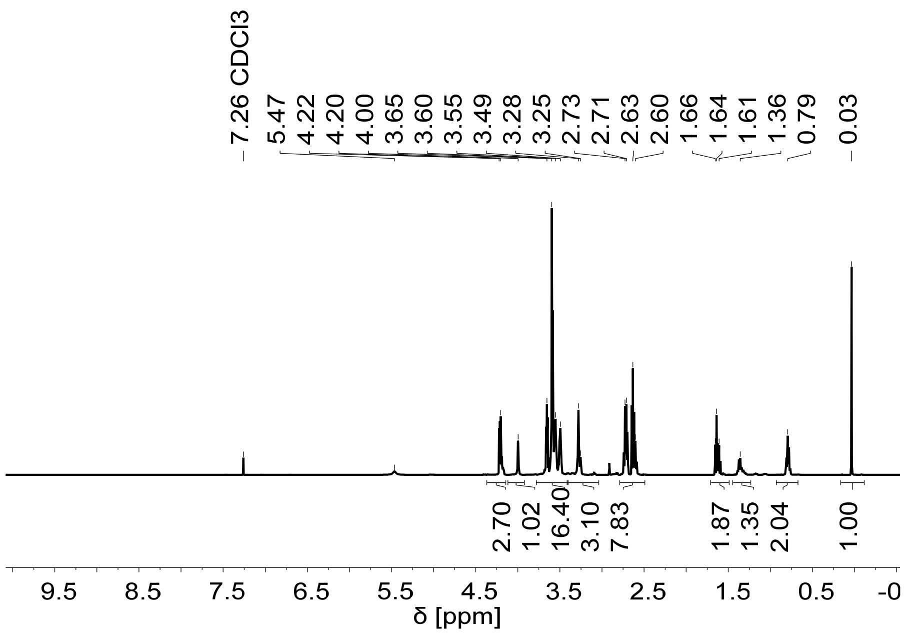

Figure S 26. ^{1}H NMR spectrum of ETTMP$_{700}$ in $CDCl_3$ at 500 MHz with OMCTS as internal standard.

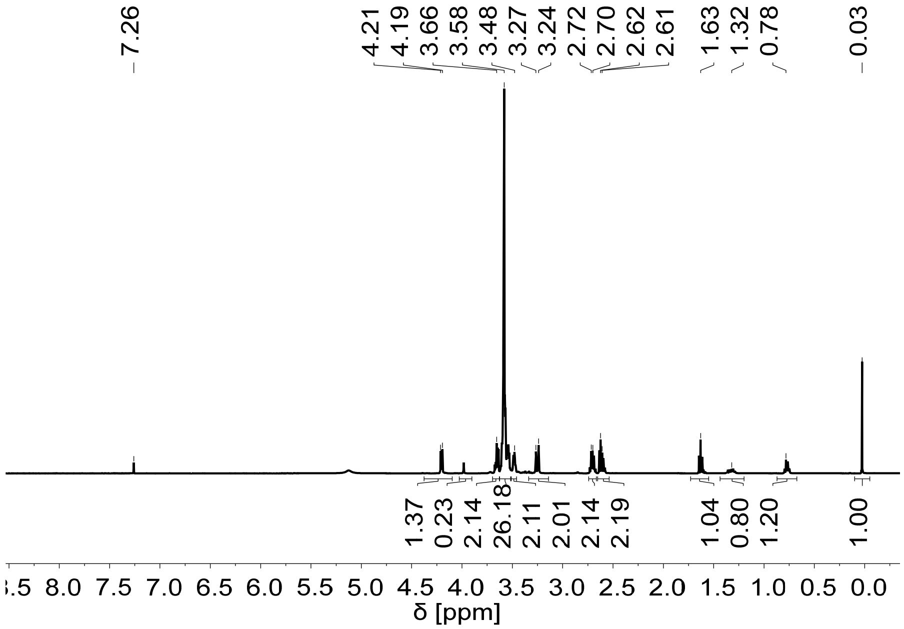

Figure S 27. ^{1}H NMR spectrum of ETTMP$_{1300}$ (aged) in $CDCl_3$ at 500 MHz with OMCTS as internal standard.

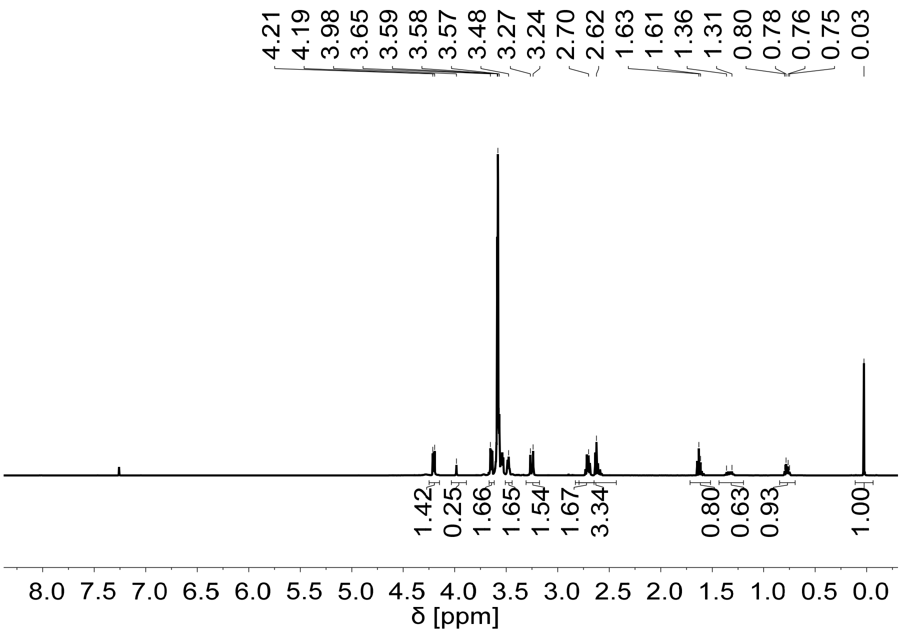

Figure S 28. ^{1}H NMR spectrum of ETTMP$_{1300}$ (fresh) in $CDCl_3$ at 500 MHz with OMCTS as internal standard.

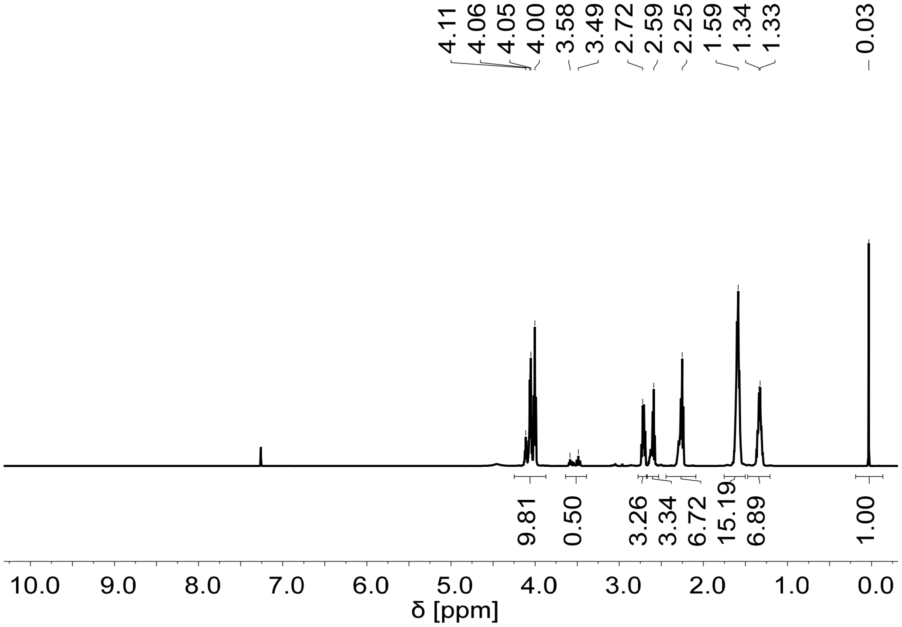

Figure S 29. ^{1}H NMR spectrum of PCL4MP in $CDCl_3$ at 500 MHz with OMCTS as internal standard.

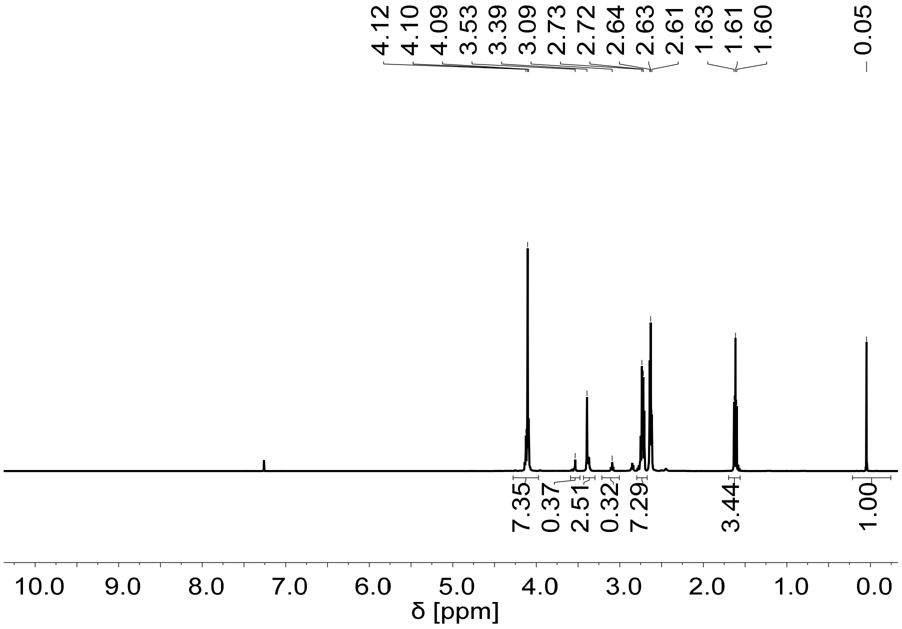

Figure S 30. ^{1}H NMR spectrum of DHMP in $CDCl_3$ at 500 MHz with OMCTS as internal standard.

Table S 8 Quantification of macromolecular thiol compounds presents by theoretical estimation based on ideal molecular structure, classical thiol analysis based on Ellman's assay, ^{1}H NMR and after derivatization by ^{31}NMR giving chemical shift (δ_{31P}).

**Compound**		**δ_{31P} [ppm]**	***c* [mmol·g^{-1}]**		
			theoretical estimation	Classical assays a/b/c	^{31}P NMR
PEG_{2000} dithiol		215.96	1.00	0.97[a]	0.94
PEG_{3000} dithiol		215.96	0.67	0.52[a]	0.50
PEG_{8000} dithiol		215.96	0.25	0.21[a]	0.20 ± 0.01
$ETTMP_{700}$		215.90	4.29	2.89[b]/2.79[c]	3.18
$ETTMP_{1300}$	aged	215.90	2.3	2.28[b]/1.74[c]	1.18
	fresh			2.30[b]/1.88[c]	1.47
PCL4MP		216.10	2.96	3.51[b]	1.70
DHMP		215.90	7.69	7.06[b]/7.42[c]	5.72

[a] Ellman's assay; [b] thiol α-CH in ^{1}H NMR; [c] Ester α-CH in ^{1}H NMR

5.7 Reproducibility and Precision of Thiol Quantification by ^{31}P NMR

Five individual measurements were prepared for each test series, and their concentrations were determined using the TMDP based ^{31}P NMR protocol. Based on the individual measured values, the mean and standard deviation were calculated. Results are reported as the mean value together with the range of one standard deviation (mean ± SD).

Table S 9. Reproducibility of thiol quantification for PEG_{8000} dithiol and $ETTMP_{700}$ Determined by ^{31}P NMR.

compound	*m* [mg]	δ_{31P} [ppm]	*Integral*	*c*(SH) [mmol·g^{-1}]			Figure
PEG_{8000} dithiol	34.2	215.9	0.80	0.21			S20
	14.1		0.29	0.21			S31
	12.9		0.26	0.20			S32
	16.0		0.33	0.21			S33
	20.2		0.39	0.20			S34
				SH	OH	COOH	
$ETTMP_{700}$	33.2	215.8	10.55	3.18	0.03	0.06	S21
	46		15.43	3.35	0.36	0.21	S35
	20.4		6.73	3.30	0.43	0.25	S36
	22.3		7.52	3.37	0.29	0.16	S37
	18.5		6.06	3.28	0.21	0.10	S38

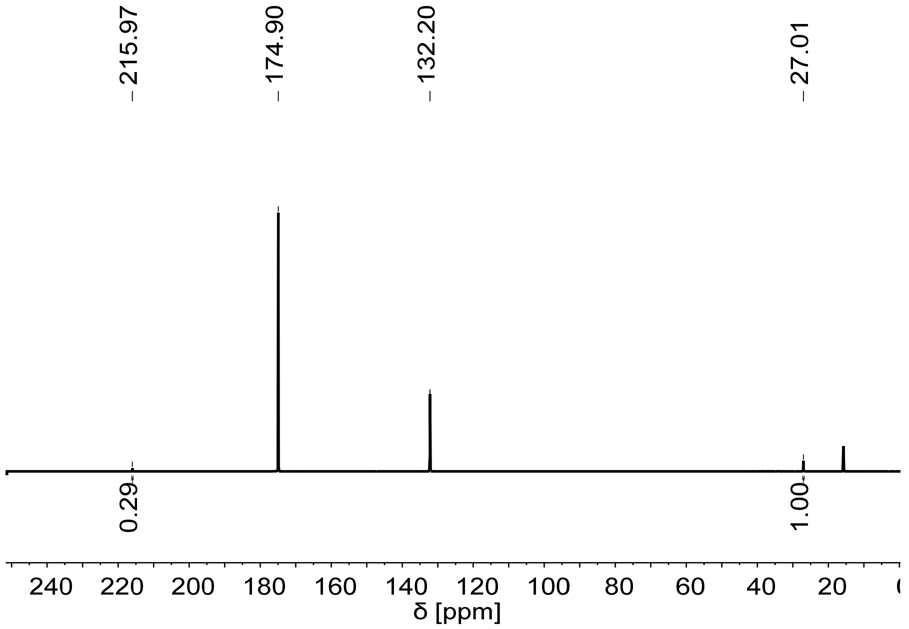

Figure S 31. ^{31}P NMR spectrum of PEG_{8000} dithiol phosphitylated with TMDP in $CDCl_3$/pyridine (1/1.6, v/v) at 202 MHz.

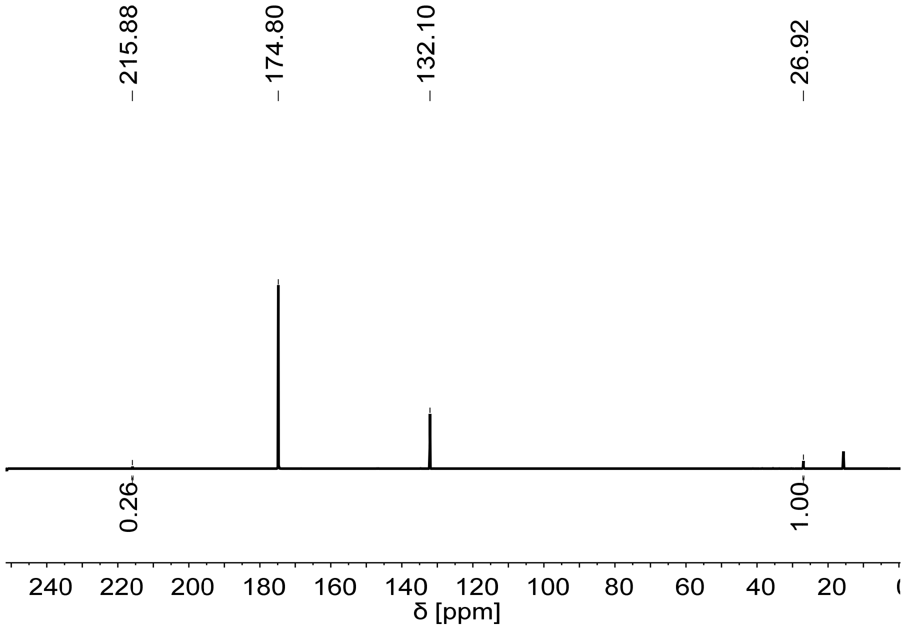

Figure S 32. ^{31}P NMR spectrum of PEG_{8000} dithiol phosphitylated with TMDP in $CDCl_3$/pyridine (1/1.6, v/v) at 202 MHz.

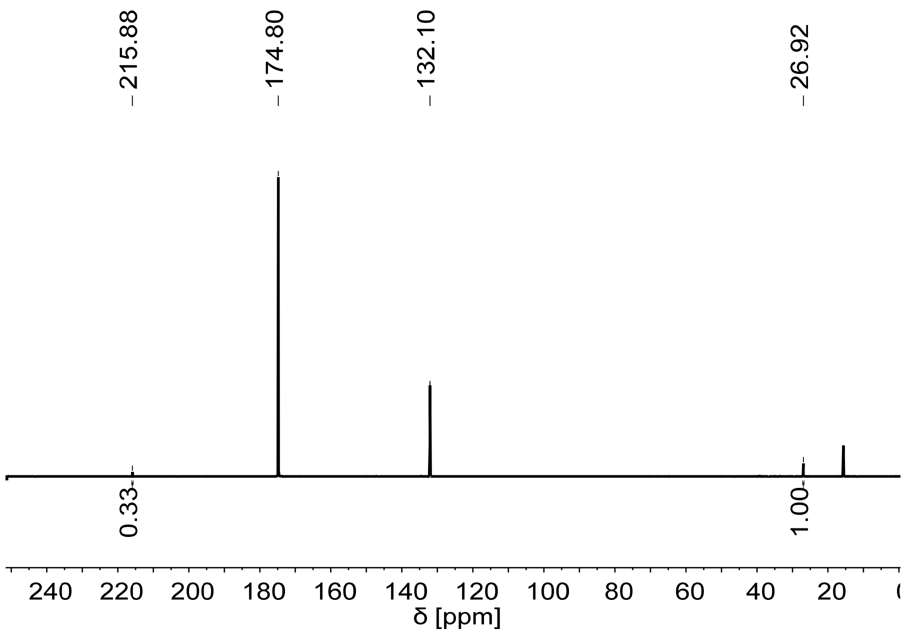

Figure S 33. ^{31}P NMR spectrum of PEG_{8000} dithiol phosphitylated with TMDP in $CDCl_3$/pyridine (1/1.6, v/v) at 202 MHz.

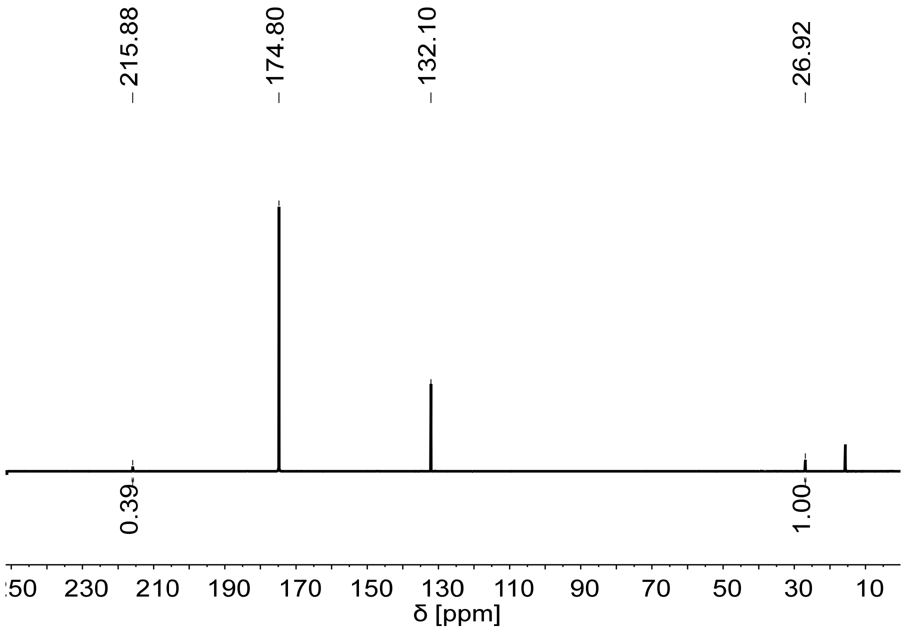

Figure S 34. ^{31}P NMR spectrum of PEG_{8000} dithiol phosphitylated with TMDP in $CDCl_3$/pyridine (1/1.6, v/v) at 202 MHz.

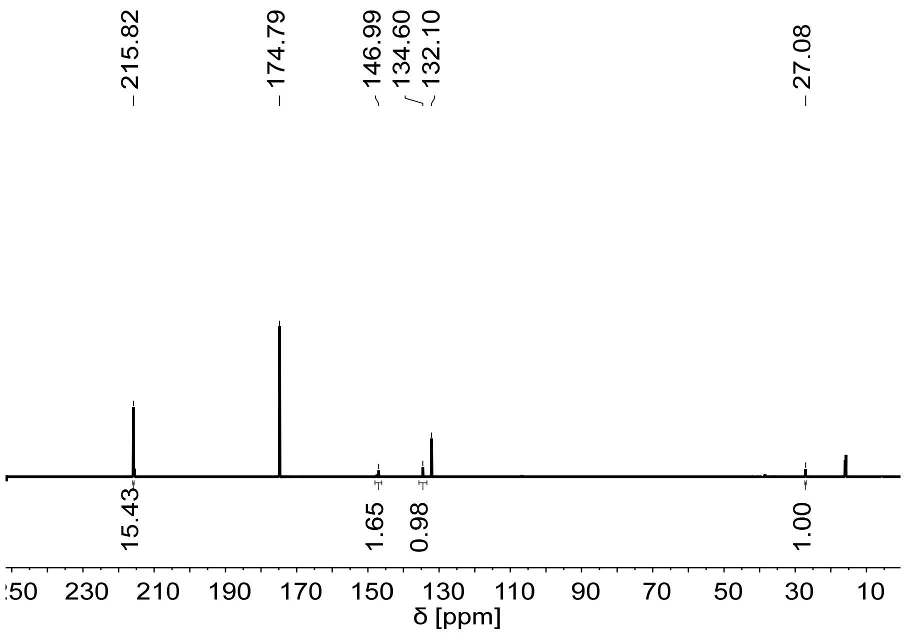

Figure S 35. ^{31}P NMR spectrum of ETTMP700 phosphitylated with TMDP in CDCl3/pyridine (1/1.6, v/v) at 202 MHz.

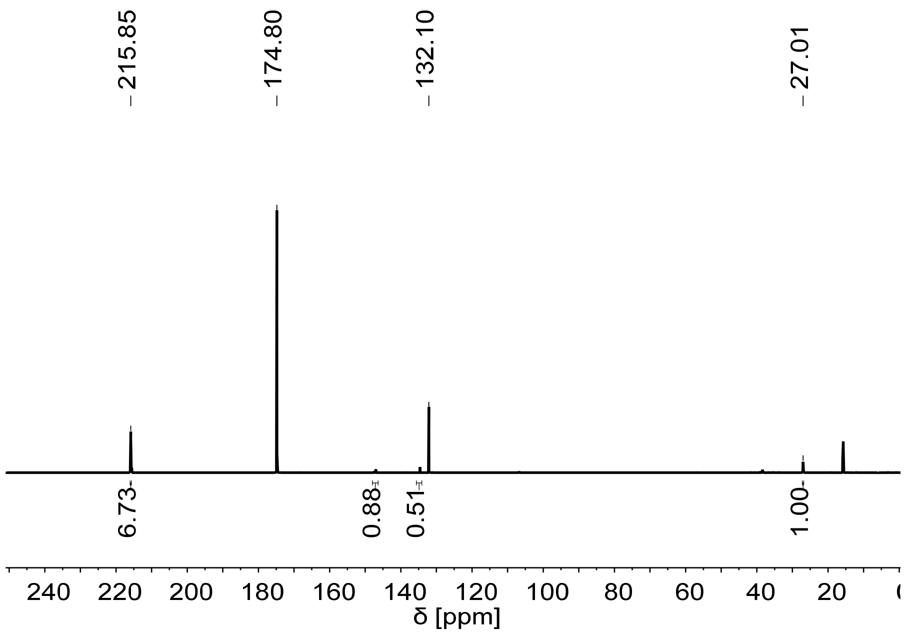

Figure S 36. ^{31}P NMR spectrum of ETTMP700 dithiol phosphitylated with TMDP in CDCl3/pyridine (1/1.6, v/v) at 202 MHz.

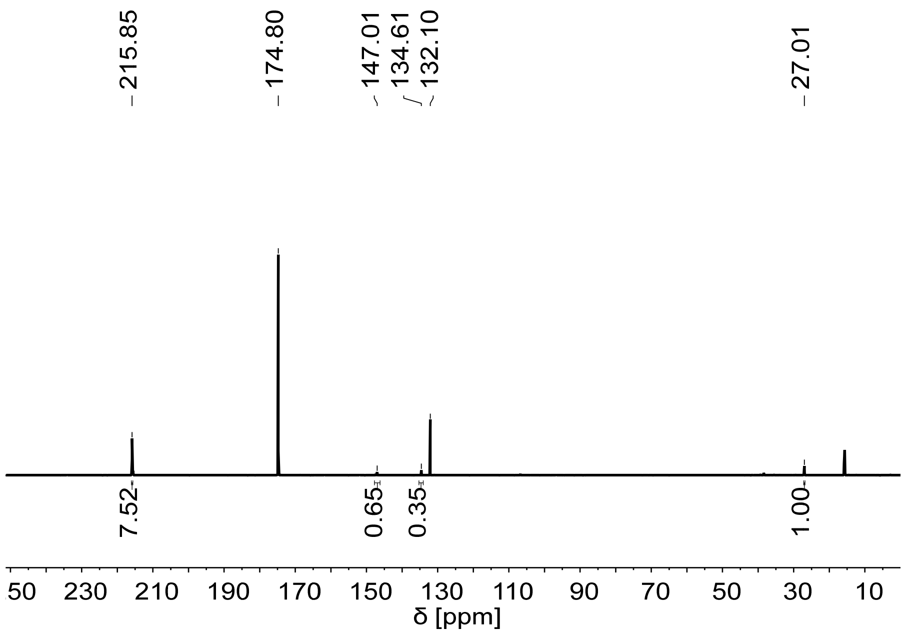

Figure S 37. ^{31}P NMR spectrum of ETTMP$_{700}$ dithiol phosphitylated with TMDP in $CDCl_3$/pyridine (1/1.6, v/v) at 202 MHz.

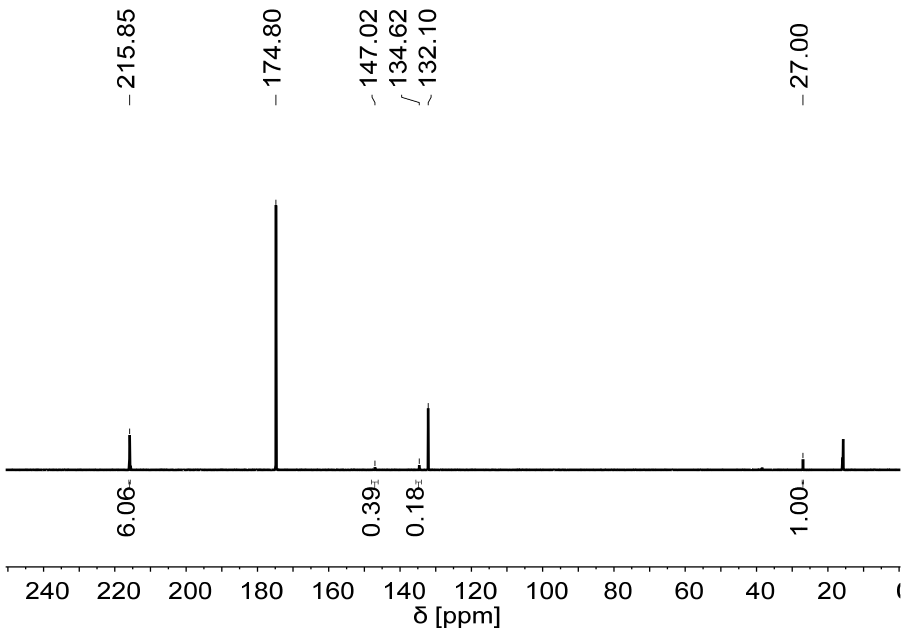

Figure S 38. ^{31}P NMR spectrum of ETTMP$_{700}$ dithiol phosphitylated with TMDP in $CDCl_3$/pyridine (1/1.6, v/v) at 202 MHz.

5.8 Analysis of RAFT dithiol

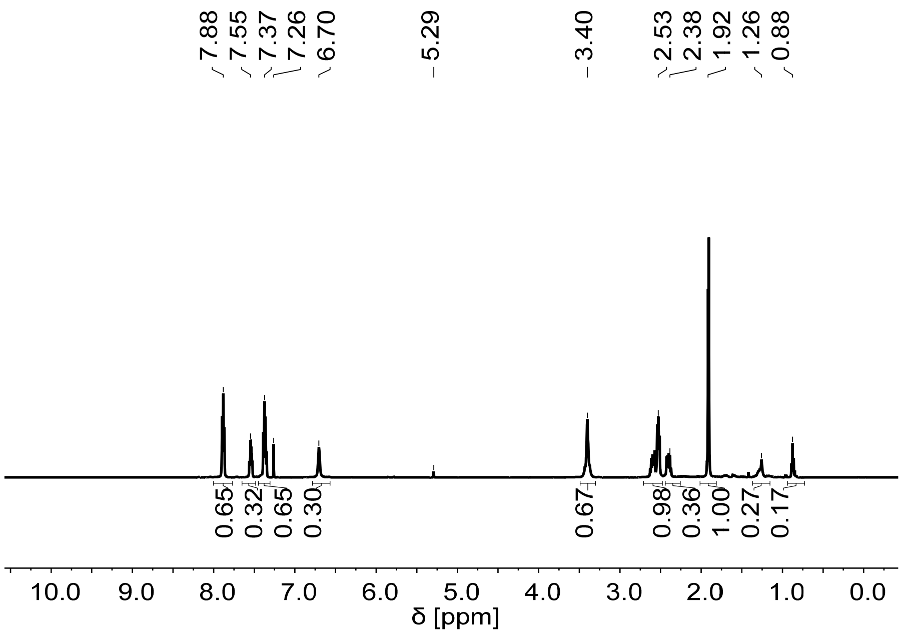

Figure S 39. ^{1}H NMR Spectrum of Bis(CTA) in $CDCl_3$ at 500 MHz.

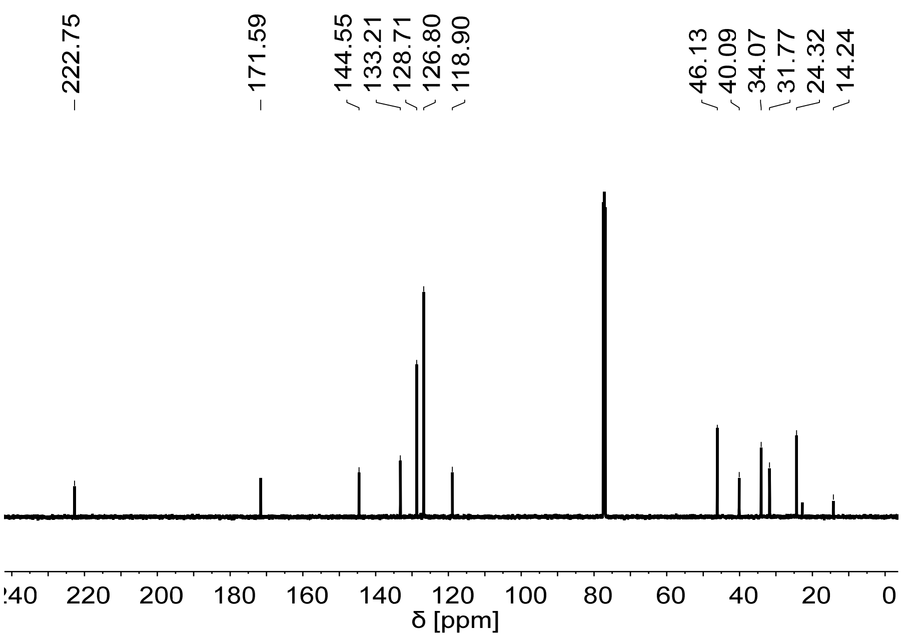

Figure S 40. . ^{13}C NMR Spectrum of Bis(CTA) in $CDCl_3$ at 126 MHz.

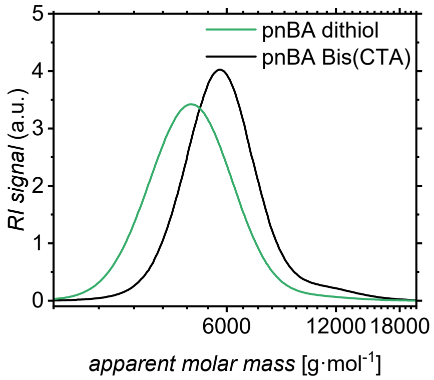

Figure S 41. GPC trace of RAFT-derived pnBA Bis(CTA) (green) and pnBA dithiol (black) recorded in THF using a refractive index (RI) detector.

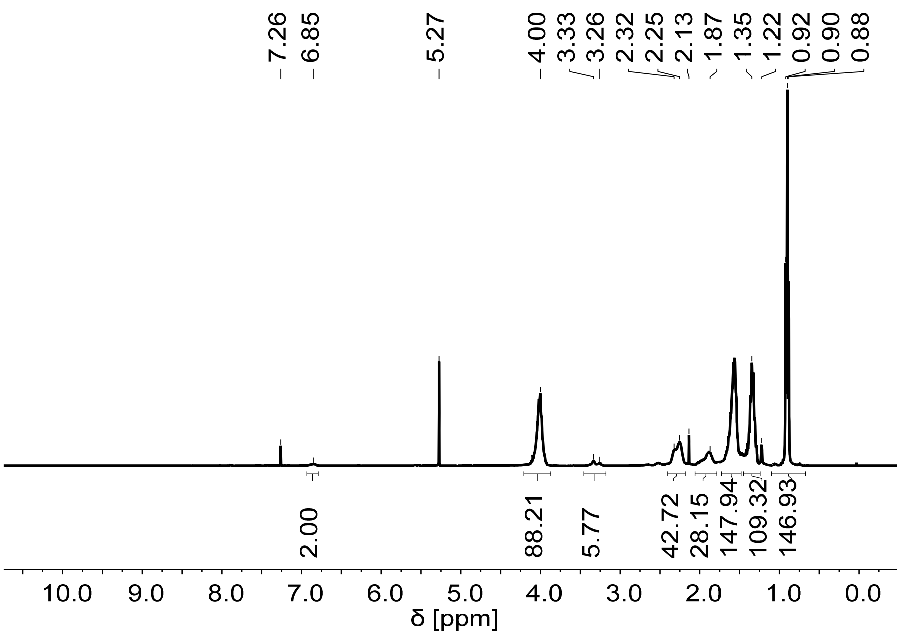

Figure S 42. ^{1}H NMR Spectrum of poly(nBA) dithiol in $CDCl_3$ at 400 MHz.

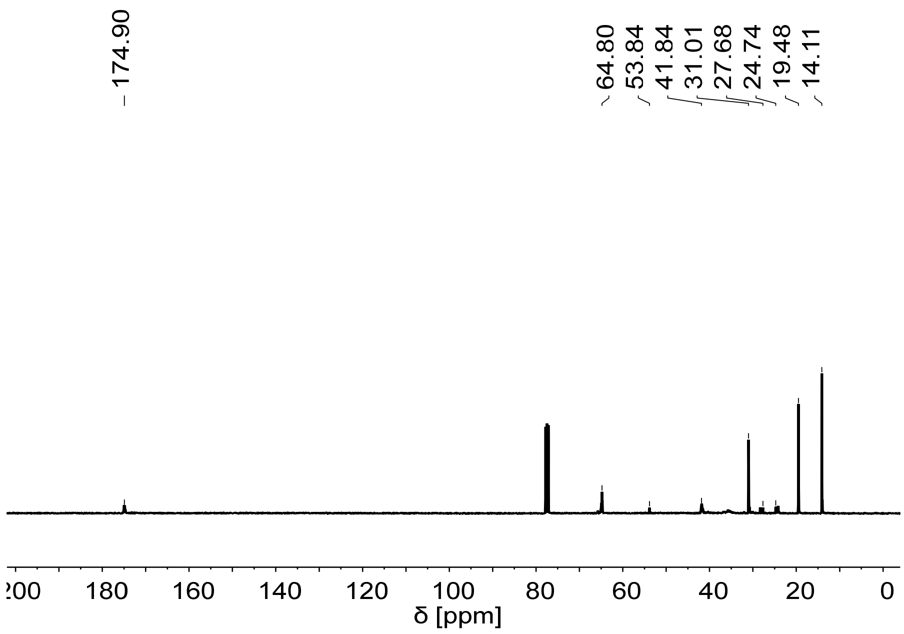

Figure S 43. ^{13}C NMR spectrum of poly(nBA) dithiol in $CDCl_3$ at 400 MHz.

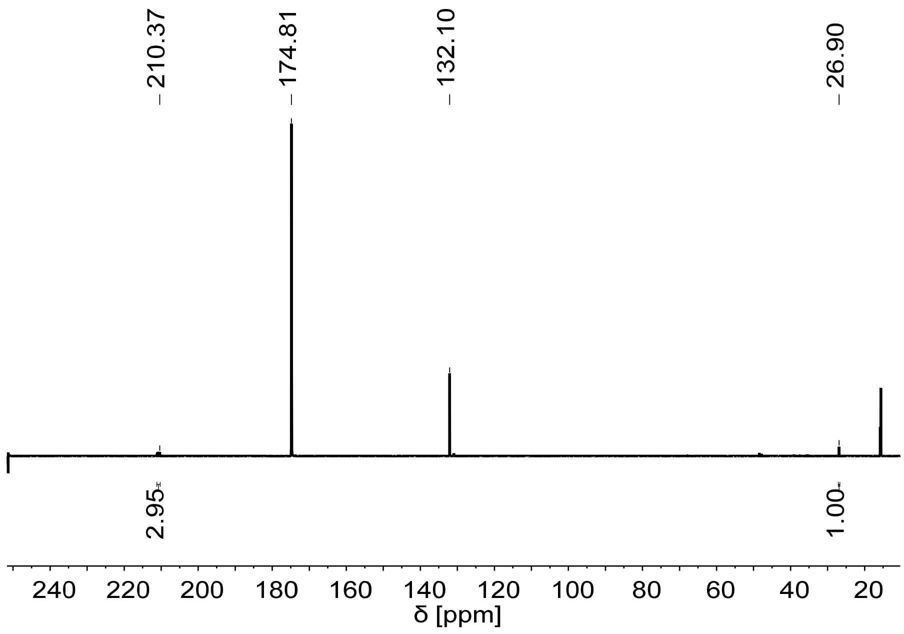

Figure S 44. ^{31}P NMR spectrum of poly(nBA) dithiol phosphitylated with TMDP in $CDCl_3$/pyridine (1/1.6, v/v) at 202 MHz. It was 122.8 mg of pnBA used.

6 References

(1) Ye, Y.-S.; Shen, W.-C.; Tseng, C.-Y.; Rick, J.; Huang, Y.-J.; Chang, F.-C.; Hwang, B.-J. Versatile grafting approaches to star-shaped POSS-containing hybrid polymers using RAFT polymerization and click chemistry. *Chem. Commun.* **2011**, *47* (38), 10656-10658.
(2) Thang, S. H.; Chong, Y. K.; Mayadunne, R. T. A.; Moad, G.; Rizzardo, E. A novel synthesis of functional dithioesters, dithiocarbamates, xanthates and trithiocarbonates. *Tetrahedron Lett.* **1999**, *40* (12), 2435-2438.
(3) Schröter, C. M.; Bangert, L. D.; Börner, H. G. Enhancing adhesion properties of commodity polymers through thiol–catechol connectivities: a case study on polymerizing polystyrene-telechelics via thiol–quinone Michael polyaddition. *ACS Macro Lett.* **2024**, *13* (4), 440-445.
(4) Meng, X.; Crestini, C.; Ben, H.; Hao, N.; Pu, Y.; Ragauskas, A. J.; Argyropoulos, D. S. Determination of hydroxyl groups in biorefinery resources via quantitative 31P NMR spectroscopy. *Nat. Protoc.* **2019**, *14* (9), 2627-2647.
